# Advanced Anode Materials of Potassium Ion Batteries: from Zero Dimension to Three Dimensions

**DOI:** 10.1007/s40820-020-00541-y

**Published:** 2020-10-28

**Authors:** Jiefeng Zheng, Yuanji Wu, Yingjuan Sun, Jianhua Rong, Hongyan Li, Li Niu

**Affiliations:** 1 Department of Materials Science and Engineering, College of Chemistry and Materials Science, Jinan University, Guangzhou, 510632 People’s Republic of China; 2 Center for Advanced Analytical Science, c/o School of Chemistry and Chemical Engineering, Guangzhou University, Guangzhou, 510006 People’s Republic of China

**Keywords:** Potassium ion batteries, Anode, Structure design, Nanomaterials, Dimensions

## Abstract

Potassium ion batteries (PIBs) with the prominent advantages of sufficient reserves and economical cost are attractive candidates of new rechargeable batteries for large-grid electrochemical energy storage systems (EESs). However, there are still some obstacles like large size of K^+^ to commercial PIBs applications. Therefore, rational structural design based on appropriate materials is essential to obtain practical PIBs anode with K^+^ accommodated and fast diffused. Nanostructural design has been considered as one of the effective strategies to solve these issues owing to unique physicochemical properties. Accordingly, quite a few recent anode materials with different dimensions in PIBs have been reported, mainly involving in carbon materials, metal-based chalcogenides (MCs), metal-based oxides (MOs), and alloying materials. Among these anodes, nanostructural carbon materials with shorter ionic transfer path are beneficial for decreasing the resistances of transportation. Besides, MCs, MOs, and alloying materials with nanostructures can effectively alleviate their stress changes. Herein, these materials are classified into 0D, 1D, 2D, and 3D. Particularly, the relationship between different dimensional structures and the corresponding electrochemical performances has been outlined. Meanwhile, some strategies are proposed to deal with the current disadvantages. Hope that the readers are enlightened from this review to carry out further experiments better.

## Highlights

- This review introduces the recent anode materials of potassium ion batteries classified into 0D, 1D, 2D, and 3D, mainly including carbon materials, metal-based chalcogenides and metal-based oxides, and alloying materials.
- The advantages, disadvantages, and optimized strategies of different dimensional anode materials are summarized.
- The relationship between different dimensional anode materials in potassium ion batteries and the corresponding electrochemical performances is outlined. And some strategies are proposed to deal with the current disadvantages of potassium ion batteries.

## Graphical abstract

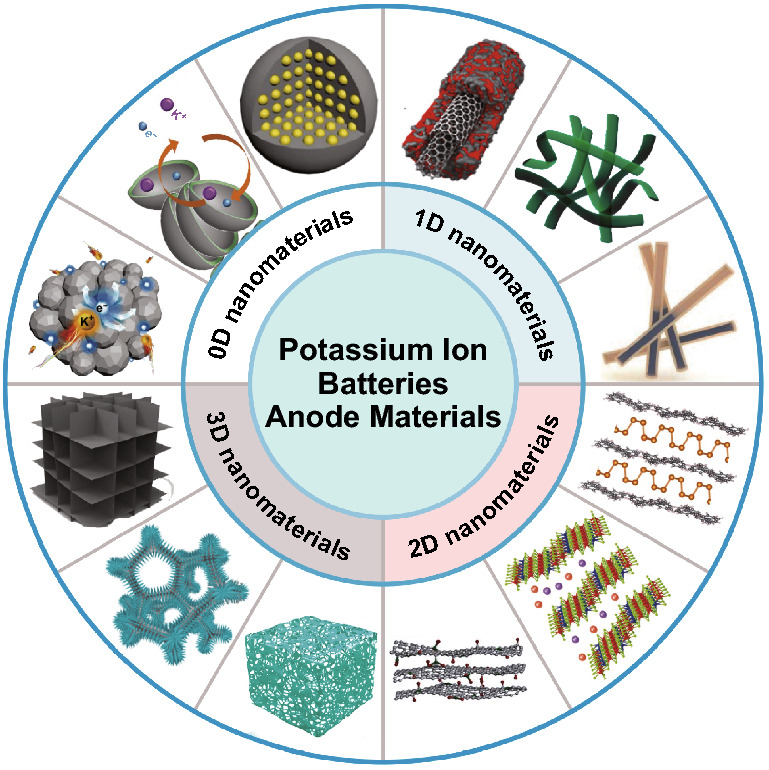

## Introduction

In 2004, it was the first time that Ali Eftekhari proposed the prototype of PIBs [1]. Nonetheless, the later stagnation about PIBs researches was ascribed to the safety issues about the K as well as technologies about other metal ion batteries becoming increasingly popular [2]. Owing to fossil fuels used increasingly as well as the severity of global warming, renewable and sustainable energy sources have been exploiting, including solar, wind, rain, geothermal, tide, and wave energies. Nevertheless, EESs must be considered to solve the intermittent issues of clean energy, which has a great effect on storing and delivering these energy resources [3, 4]. So far, lithium ion batteries (LIBs), as a representative energy storage technology, have been widely explored for portable devices, electrical vehicles, and large-grid EESs because of high energy density and stable cycling lifespan [5]. Unfortunately, there are some barriers for LIBs to develop sustainably, such as challenging lithium (Li) recovery, uneven distribution of Li, and increasing cost [6–8]. Accordingly, it is necessary to search for alternative rechargeable batteries’ technologies. Based on cost and resource considerations, scientists have put a lot of effort into developing a series of non-lithium ion batteries, including sodium ion batteries (SIBs) [9], PIBs [10], magnesium ion batteries (MIBs) [11], zinc ion batteries (ZIBs) [12], aluminum ion batteries (AIBs) [13] and so on [14]. Since zinc, magnesium, and aluminum are less active than lithium, they could be used as anode materials for metal ion batteries [15–17]; especially, magnesium and aluminum anodes do not even form dendrites, so their corresponding ion batteries are able to meet the safe requirement [18, 19]. In addition, commercial LIBs mainly use graphite with a theoretical specific capacity of 372 mAh g^−1^ as anode material, while the capacities of zinc (820 mAh g^−1^) [20], magnesium (2205 mAh g^−1^), and aluminum (2980 mAh g^−1^) anodes are much higher than that of graphite [18]. Clearly, each multivalent cation can exchange more than one electron, suggesting that if a host material can store the same number of cations, the capacities of multivalent ion cells are several times that of monovalent ion cells. Therefore, these multivalent ion batteries may have higher energy densities. Furthermore, the ion radii of zinc ions (0.74 Å) [12], magnesium ions (0.72 Å) [21], and aluminum ions (0.535 Å) [21] are relatively small, having low trends to damage the structure of host material. However, although these multivalent metal ion batteries have various irreplaceable advantages, they also face some intractable problems. First of all, the surface charge density of these multivalent metal ions is relatively high, resulting in greater mutual repulsion between cations and greater interaction between cations and host materials, which is not conducive to high capacity and high rate performance for batteries [22]. Besides, the potentials of Zn/Zn^2+^ (− 0.76 V vs. SHE) [23], Mg/Mg^2+^ (− 2.37 V vs. SHE) [14], Al/Al^3+^ (− 1.76 V vs. SHE) [13] are much higher than that of Li/Li^+^ (− 3.04 V vs. SHE), indicating that it is difficult for these batteries to obtain high operating voltages. There are many other problems that hinder their development. For instance, the zinc anode is still plagued by dendrites problem [24] and the electrolyte used in MIB is not ideal [14, 25, 26]. Compared with MIBs, ZIBs, and AIBs, both the working principle and the electrode materials and electrolytes used in SIBs and PIBs are similar to those of LIBs, for the reason that sodium, potassium, and lithium have similar physical and chemical properties [27]. It is expected that PIBs and SIBs have fewer obstacles for commercial applications.

**Fig. 1:**
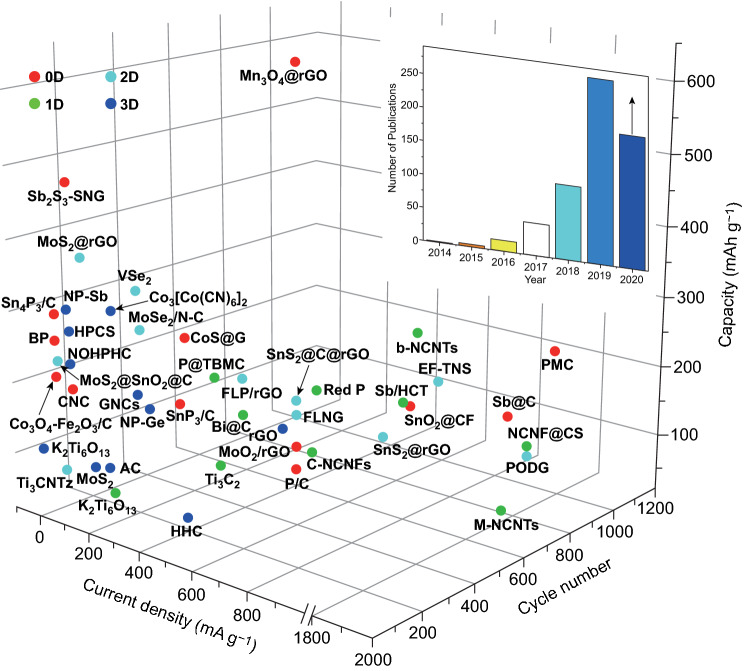
Cycle capacity of various PIBs anode materials reported recently. Sb_2_S_3_-SNG [38]; Co_3_O_4_-Fe_2_O_3_/C [39]; black phosphorus = BP [33]; Sn_4_P_3_/C [40]; CoS@G [41]; CNC [42]; PMC [43]; Sb@C [44]; P/C [45]; MoO_2_/rGO hollow sphere composites = MoO_2_/rGO [46]; Mn_3_O_4_@rGO [47]; SnP_3_/C [48]; SnO_2_ nanoparticles anchored on carbon foam = SnO_2_@CF [49]; P@TBMC [50]; NCNF@CS [51]; alkalized Ti_3_C_2_ MXene nanoribbons = Ti_3_C_2_ [52]; chitin-derived NCNFs = C-NCNFs [53]; bamboo-like NCNTs = b-NCNTs [54]; MOF-derived NCNTs = M-NCNTs [55]; red P [56]; segment-like Sb nanorod encapsulated in hollow carbon tube = Sb/HCT [57]; K_2_Ti_6_O_13_ [58]; Bi@C [59]; Ti_3_CNTz [60]; MoS_2_@rGO [61]; MoS_2_@SnO_2_@C [62]; graphene-like VSe_2_ nanosheets = VSe_2_ [63]; Few-layered SnS_2_ nanosheets supported on rGO = SnS_2_/rGO [64]; FLNG [65]; PODG [66]; MoSe_2_/N–C [67]; EF-TNS [68]; FLP/rGO [69]; MoS_2_ [70]; K_2_Ti_6_O_13_ [71]; NP-Sb [72]; NP-Ge [73]; activated carbon = AC [74]; Co_3_[Co(CN)_6_]_2_ [75]; HPCS [76]; N/O co-doped mesoporous carbon octahedrons = NOHPHC [77]; rGO aerogel = rGO [78]; GNCs [79]; honeycomb hard carbon = HHC [80]. And the inset about number of articles obtained from Web of Science on PIBs anode materials and based on the corresponding Topic about potassium ion battery, potassium ion storage, potassium storage, and anode (accessed: July 1, 2020)

PIBs aroused remarkable attentions again in 2015 and increasing researches have been publishing since then (the inset of Fig. 1). Firstly, PIBs possess similar rocking-chair operating principle compared with LIBs, which provides a favorable foundation for the studies of PIBs [28, 29]. Secondly, PIBs with lower price and sufficient resources are suitable for EESs [30]. Thirdly, the standard redox potential versus SHE of K^+^/K (− 2.93 V) is not only even comparable to that of Li^+^/Li (− 3.04 V) but also lower compared with that of Na^+^/Na (− 2.71 V); thus, PIBs are beneficial to produce higher operating voltages [31, 32]. Meanwhile, the lowest potential versus SHE of K^+^/K (− 2.88 V) is compared with that of Na^+^/Na (− 2.56 V) and Li^+^/Li (− 2.79 V) in some non-aqueous electrolytes like propylene carbonate solvent, which makes PIBs benefit from wider potential window to achieve high energy density [2, 33, 34]. Besides, Okoshi et al. indicated that weaker Lewis acidity and low K-ion desolvation energy brought about the smaller Stokes radius of solvated ions and the low interfacial reaction resistance, respectively, which made K electrolytes possess higher conductivity compared with Li as well as sodium (Na) electrolytes [35]. In addition, the commercialized graphite has been successfully explored for PIBs anode and the theoretical capacity is around 279 mAh g^−1^, but it is not suitable for SIBs [28, 29, 31]. Furthermore, the unwanted K-metal deposition on the surface of anode may be hindered due to the insertion potential versus K^+^/K of K-ion (0.2 V), which is beneficial for improving safety in terms of operation [32]. Moreover, compared with Li metal (180.54 °C) and Na metal (≈ 98 °C), K-metal lower melting point (63.38 °C) makes the dendritic K melting to provide a secure capability under an appropriate temperature [10, 36, 37]. It is worth mentioning that Cu foil can be replaced with Al foil as current collectors in anode electrode of PIBs without forming Al-K alloy, cutting down the batteries’ production expenses [29]. Given the above-mentioned advantages, PIBs are promising alternative batteries for LIBs. To date, the reported PIBs anode materials mainly contain carbon materials (e.g., carbon nanotubes (CNTs), graphene, and graphite), MCs (e.g., MoS_2_ and MoSe_2_), MOs (e.g., MoO_2_ and SnO_2_), and alloying materials (e.g., antimony (Sb), tin (Sn), bismuth (Bi), germanium (Ge), and phosphorous (P)) (Fig. 1).

Accordingly, lots of anode materials have been reported, but it is still necessary to well comprehend how to select suitable anode materials so as to make it convenient to search for the related materials. It is known that alkali ion batteries (LIBs, SIBs, and PIBs) have similar electrochemical processes due to their similar features. And then, the practical application of alkali ion batteries can be influenced because of the choice of suitable anode materials based on selection principle. Therefore, the selection principle of LIBs, SIBs, and PIBs should be considered about several aspects in detail: (1) The materials can be regarded as anode materials, depending on whether the material can react with alkali ions and the corresponding theoretical capacity; (2) The structural stability and durability should also be considered because the robust structures can better tolerate with the stress variation during long cycles; (3) The operability of the materials is also one of the considered factors, so the materials affected easily by environmental conditions like oxygen should be excluded; (4) The cost of materials also affects the practical application of batteries; (5) The recyclability of electrode materials should be paid more attention to make good use of resources and protect environment. Therefore, based on aforementioned selection principle, the anode materials can be better chosen. However, it does not mean that all anode electrodes used in LIBs can be successfully used in SIBs and PIBs, although LIBs, SIBs, and PIBs have similarities in the energy storage mechanism. For examples, the aforementioned graphite can be used as the commercial LIBs electrode materials but not suitable for Na^+^. This phenomenon is possibly attributed to two main reasons: (1) Ionic insertion can be affected because of different storage reactions between ions and the corresponding active materials and (2) Different ion radii are necessary to search for related different material accommodating their ionic size, such as Li^+^ (0.76 Å), Na^+^ (1.02 Å), and K^+^ (1.38 Å). So some anode materials may be conducive to hold Li^+^ but not in favor of containing large K-ion.

The aforementioned electrode materials have exhibited distinct electrochemical performances due to their unique features. Meanwhile, it is well known that the materials properties will be affected due to different dimensional structures. Zero-dimensional (0D) nanomaterials can promote ionic adsorption and mitigate stress variation owing to surface effect and small size effect. As 0D nanomaterials, SnO_2_ nanoparticles anchored on carbon foam not only facilitated electrolyte penetration but also boosted K-ion transport, which realized the cycle capacity of 231.7 mAh g^−1^ at 1 A g^−1^ after 400 cycles as well as the rate capacity of 143.5 mAh g^−1^ at 5 A g^−1^ [49]. Furthermore, one-dimensional (1D) nanomaterials with high length-to-diameter ratio are beneficial to enhance the electronic and ionic transfer and provide high mechanical robustness. For example, owing to unique architecture, nitrogen (N)-doped carbon nanofibers (CNFs) as electrode materials in PIBs exhibited outstanding performances, delivering a capacity of 146 mAh g^−1^ at 2 A g^−1^ after 4000 cycles [81]. In addition, two-dimensional (2D) nanomaterials are also capable of improving ionic adsorption and facilitating ionic diffusion owing to high surface area as well as tunable interlayer spacing. As one of the typical 2D materials, few-layer bismuthene explored for PIBs anode materials could facilitate electrolyte infiltration, boost K-ion transfer, and buffer volumetric expansion in the course of charge and discharge process, so a capacity of more than 200 mAh g^−1^ was delivered at 20 A g^−1^ over 2500 cycles [82]. As for three-dimensional (3D) nanomaterials, with high mechanical strength and interconnected structures, they can effectively tolerate stress variation and make largely electrode contact with electrolyte as well as promote electronic conductivity. In 2019, 3D reduced graphene oxide (rGO) aerogel was fabricated to enhance K-ion transfer, improving rate performance (92 mAh g^−1^ at 6.7 C) as well as cycle performance (267 mAh g^−1^ at C/3 after 100 cycles) [78].

**Fig. 2:**
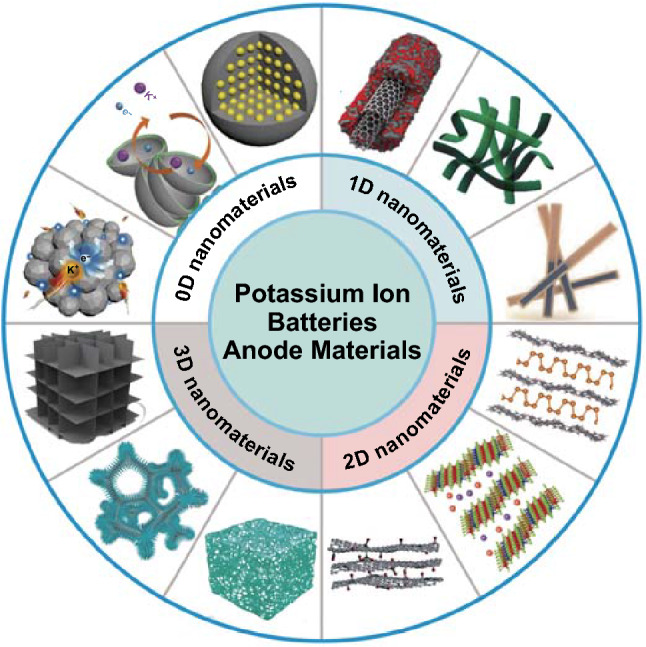
Schematic diagram of various 0D–3D PIBs anode materials. Reproduced with permission from Ref. [42, 43, 68, 89]. Copyright 2018 (2019), John Wiley and Sons; from Ref. [66, 90, 91]. Copyright 2017 (2019, 2020), Royal Society of Chemistry; from Ref. [50]. Copyright 2018, Elsevier; from Ref. [69, 72, 92, 93]. Copyright 2017 (2018, 2019), American Chemical Society

In this review, 0D, 1D, 2D, and 3D nanomaterials about the recent developed PIBs anode materials will be introduced (Fig. 2), mainly concentrating on carbon materials, MCs and MOs, and alloying materials. Among them, carbon materials with the advantage of low price are attributed to its abundant sources, which is beneficial for practical applications. In addition, the existing technology can be used to prepare carbon nanomaterials, making the synthesis process convenient. And then, the structures of carbon materials are relatively stable and durable, which helps to keep structural stability and improve the long life of the corresponding electrode during charging and discharging. As for MCs and MOs, they have high capacity and it is easy to synthesize. Besides, alloying materials not only possess high theoretical capacity, such as Sb (660 mAh g^−1^) [83] and Bi (385 mAh g^−1^) [83], but also can react with K-ion under low potentials (~ 0.1–0.8 V vs. K^+^/K) [72, 84–88]. Additionally, similar to MCs and MOs, alloying materials can be prepared by some easy synthesis methods, so it is convenient to obtain the corresponding products [2]. According to these advantages, alloying materials can be considered as anode materials of PIBs. Furthermore, the structural design and the corresponding electrochemical performances of different dimensional anode materials will be summarized. To date, the capacities of 0D–3D carbon materials have varied from 200 to 600 mAh g^−1^. Moreover, MCs and MOs with 0D–3D structures have delivered the capacities with 50–600 mAh g^−1^. In addition, the capacities of 0D–3D alloying materials varied from 300 to 1000 mAh g^−1^ have been studied [36]. Meanwhile, some practical strategies about solving some challenging issues will be proposed.

## Zero-Dimensional Nanomaterials for PIBs

0D nanomaterials are defined as 1 nm to 100 nm in three dimensions. So far, 0D nanomaterials have various types, including quantum dots, nanoparticles, nanospheres, nanocages, core–shell structures, and so forth. Typically, these 0D nanomaterials have been widely studied in energy storage field due to structural features and properties including surface effect, small size effect, and so on; especially, the small size of 0D nanomaterials possesses large surface area, providing sufficient sites for ionic adsorption [94, 95]. Additionally, the stable structure plays a crucial role in buffering large volume expansion, like hollow 0D nanomaterials. Therefore, 0D nanomaterials are beneficial for anode materials of PIBs to keep contact with electrolyte and restrict volumetric variation. However, the electrochemical performances will be affected due to 0D nanomaterials with easier self-aggregation. Thus, it is better to be composited with other materials to inhibit aggregation for anode materials. Next, the relationship between 0D nanomaterials and the corresponding electrochemical performances will be elaborated in detail. In addition, the initial Coulombic efficiency (C.E.), rate performances, and cycle properties of recent reported 0D anode materials of PIBs are summarized in Table 1.

**Table 1:** Comparison of the state-of-the-art performances of 0D anode materials in PIBs

Materials	Initial C.E. (%)	Rate capacity (mAh g^−1^) at the current density (mA g^−1^)	Cycle capacity (mAh g^−1^) at the current density (mA g^−1^) (cycle number)	References
Sb_2_S_3_-SNG	69.7	340 at 1000	480.078 at 50 (100)	[38]
Co_3_O_4_-Fe_2_O_3_/C	54	–	220 at 50 (50)	[39]
BP	60	300 at 2000	270 at 50 (50)	[33]
Sn_4_P_3_/C	–	221.9 at 1000	307.2 at 50 (50)	[40]
Sb@C	75.8	127 at 2000	160 at 1000 (800)	[44]
Sb@PC	46.2	200 at 2000	90 at 500 (200)	[96]
P/C	50.3	90 at 500	71.5 at 500 (500)	[45]
ZNP/C	58.5	46 at 2000	145 at 500 (300)	[97]
Sb@NPMC	50	161 at 1000	130 at 1000 (1500)	[98]
MoO_2_/rGO	51.6	176.4 at 500	104.2 at 500 (500)	[46]
Co_0.85_Se-QDs/C	61.8	220 at 2000	228 at 1000 (500)	[99]
S,N co-doped thin carbon	–	64 at 4000	65 at 2000 (900)	[100]
Mn_3_O_4_@rGO	66	95 at 10,000	635 at 500 (500)	[47]
Fe_x_O@NFLG	46	176 at 5000	140 at 5000 (5000)	[101]
Titanium oxynitride nanoparticles/carbon	–	72 at 1600	150 at 200 (1250)	[102]
SnP_3_/C	58.8	221.8 at 1200	225 at 500 (80)	[48]
SnO_2_@3D PC	13.99	144.6 at 2000	270.3 at 100 (200)	[103]
VN-QDs/CM	72.9	152 at 2000	215 at 500 (500)	[104]
Co_9_S_8_/N-C@MoS_2_	89.1	50 at 1000	100 at 1000 (100)	[105]
Carbon-coated mesoporous Co_9_S_8_ nanoparticles supported on rGO	59.5	215.1 at 5000	210.8 at 1000 (1200)	[106]
SnO_2_@CF	44.43	143.5 at 5000	231.7 at 1000 (400)	[49]

### Zero-Dimensional Carbon Materials

**Fig. 3:**
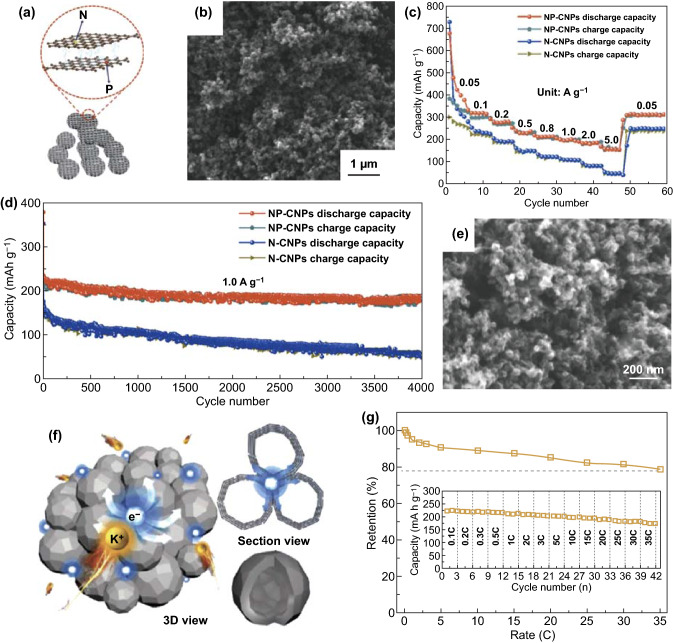
NP-CNPs’ **a** schematic diagram, **b** SEM image, **c** as well as N–CNPs’ rate capacities in the range of 0.05–5.0 A g^−1^, **d** long cycle capacities at 1.0 A g^−1^ over 4000 cycles. Reproduced with permission from Ref. [107]. Copyright 2019, American Chemical Society. **e** SEM image of CNC. **f** Schematic illustration of CNC. **g** Discharge capability retention corresponding to rate performance (inset). Reproduced with permission from Ref. [42]. Copyright 2018, John Wiley and Sons

0D carbon materials with shorter ionic diffusion pathway are advantageous to reduce transport resistances. Accordingly, a few studies about nanoparticles have recently been reported so as to improve the electrochemical behavior. Carbon nanoparticles with N/P co-doping and expanded interlayer (NP-CNPs) were fabricated for the PIBs electrode materials [107]. The synergistic effect of nano-size and P/N-co-doping helped to obtain good electrochemical performances. Then, the NP-CNPs electrode had a capacity of 157 mAh g^−1^ at 5.0 A g^−1^ (Fig. 3c). In addition, a capacity of 190 mAh g^−1^ was obtained at 1.0 A g^−1^ after 4000 cycles (Fig. 3d). According to experimental test, the nanoparticles with the expanded interlayer and uniform ultrafine nanoparticles were exhibited (Fig. 3a, b). Obviously, the morphology of nanoparticles and its expanded interlayer could effectively improve the electrochemical performances, which not only provided shorter K-ion transfer path as well as improved electrical conductivity, but also enhanced adsorption capability toward K-ions.

Besides, novel hollow structures may be advantageous for accommodating the volumetric changes compared with solid nanoparticles. For example, graphite has been wildly utilized but still suffered from large volumetric variation during charge and discharge process as PIBs electrode. Although some progress has been achieved in terms of regulating structure including polynanocrystalline graphite, activated carbon, and expanded graphite, it is still challenging to ensure the robust stability of graphite during long cycling process [74, 108–110]. It is worthwhile that hollow nanocages have gradually been used for electrode materials. Cao et al. [42] synthesized graphitic carbon nanocage (CNC) as anode material for PIBs by taking advantage of Ketjen carbon black. From Fig. 3e, f, it can be clearly seen that the CNC possessed interlinked structural appearance, which benefited fast electronic transfer. As for the electrochemical performances, reversible capacities of the CNC electrode were 221.5 mAh g^−1^ as well as 175 mAh g^−1^ at 0.1 and 35 C, respectively (Fig. 3g). As expected, the electrochemical behavior of the obtained electrode was due to its unique structure features. In detail, the cage-like structure kept the stability of structure by reducing the anisotropy and avoiding interlayer slipping. Then, the cage-like hollow interior could retain the structural integrity by accommodating volume changes well in the course of charging and discharging. Similarly to soft carbon, hollow graphitized carbon nanocages (HGCNs) were also synthesized as effective PIBs electrode materials [111]. The authors exhibited that HGCN-1000 (1000 mean the carbonization temperature of 1000 °C) with highly graphitized carbon-cage, developed porosity, and hollow structure was endowed robust structural stability and good electrochemical performances. Therefore, the capacity retention of 95.9% for the HGCN-1000 electrode was achieved at 1 A g^−1^ over 2000 cycles, which identified better cyclic stability.

Based on the above-mentioned examples, 0D carbon materials including nanoparticles and nanocages have been fabricated to effectively enhance the electrochemical performances, devoted to fast ionic transportation and high structural stability. However, compared with LIBs and SIBs, few studies about carbon dots (CDs) applied for anode materials of PIBs have been reported [112–120]. Therefore, it is necessary for further experiments to focus on novel structure for the family of 0D carbon anode materials, such as CDs, core–shell structure, and yolk shell structure.

### Zero-Dimensional MCs and MOs

MCs and MOs have proved to be a kind of hopeful PIBs electrode materials, but severe volume changes and low electrical conductivity have hindered their development. It is well known that the minimization of materials is conducive to alleviating stress changes, so the preparation of 0D MCs and MOs is an effective method. In order to solve low conductivity and agglomeration problems, MCs and MOs are usually combined with conductive materials.

**Fig. 4:**
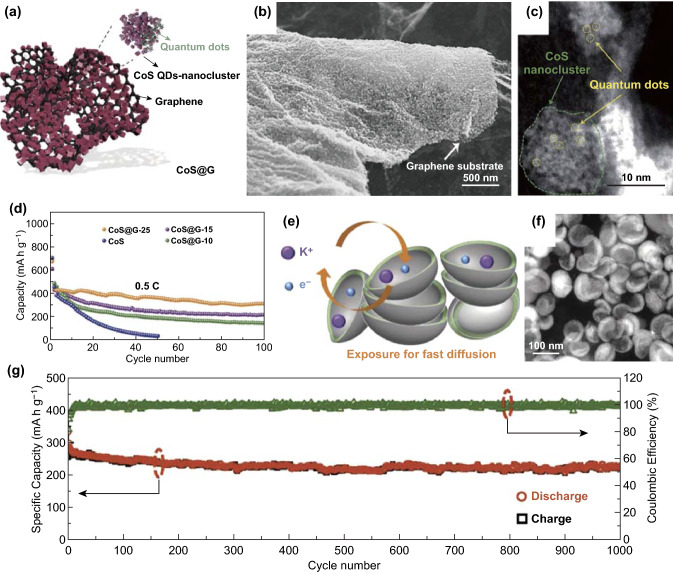
**a** Schematic illustration of CoS@G composite. **b** SEM and **c** dark field images of the obtained sample. **d** Cycling capacities of CoS@G-25 anode compared with other samples. Reproduced with permission from Ref. [41]. Copyright 2017, John Wiley and Sons. **e** Schematic diagram of PMC with fast ion and electron diffusing. **f** HAADF-STEM image of PMC. **g** Long-term cycle performance as well as (C.E.) at 1.0 A g^−1^ over 1000 cycles. Reproduced with permission from Ref. [43]. Copyright 2018, John Wiley and Sons

Quantum dots with quantum confinement are practical architectures for boosting electronic and ionic transportation. A composite about cobalt sulfide compounded with graphene (CoS@G) was synthesized for PIBs (Fig. 4a) [41]. In this composite, graphene offering landing platform for CoS quantum dots not only contributed to restraining the agglomeration of CoS quantum dots, but also improved electronic conductivity. Then, numerous smaller CoS nanoclusters were uniformly dispersed onto the graphene nanosheets, consisting of the interlinked quantum dots (Fig. 4b, c). As illustrated in Fig. 4d, the CoS@G-25 (25 means 25% graphene oxide) electrode delivered the discharge capacity of 310.8 mAh g^−1^ at 500 mA g^−1^ after 100 cycles. Moreover, the CoS@G-25 electrode’s capacities were retained 67.3% and 56.2% at 3 C and 4 C, respectively. Therefore, it is effective for novel shapes to improve the corresponding electrochemical performances. Coating is also an effective means to prevent agglomeration and buffer volume changes, such as building a core/shell structure. So Wang et al. [43] synthesized MoSe_2_/C composites with pistachio-shuck-like morphology (PMC) as PIBs anodes to improve their performances. As shown in Fig. 4e, the packing density of the unique PMC was enhanced via plane-to-plane contact. It could expedite electronic transfer as well as K-ion diffusion and retain the structural stability during the process of potassium/depotassium. Additionally, the PMC exhibited pistachio-shuck-like morphology with the diameter of around 70–90 nm (Fig. 4f). In the electrochemical test, the PMC electrode delivered 226 mAh g^−1^ at 1.0 A g^−1^ after 1000 cycles (Fig. 4g). The PMC electrode’s electrochemical performances were due to the core of MoSe_2_, the pistachio-shuck-like structure, and thin amorphous carbon shell. These structural features could facilitate K-ion transfer and buffered the volume expansion.

Besides, titanium dioxides (TiO_2_) have been utilized in LIBs and SIBs but seldom reported for PIBs. Therefore, in 2019, Fang et al. [121] synthesized a composite about TiO_2_ nanoparticles anchored on rGO for PIBs. As for this composite, the small size TiO_2_ could boost K-ion diffusion and the rGO could promote electronic transfer. So its electrochemical performances could be enhanced, which achieved the rate capacity of 107.1 mAh g^−1^ at 1000 mA g^−1^ and the cycling capacity of 88.4 mAh g^−1^ at 1000 mA g^−1^ after 1000 cycles. Nevertheless, its electrochemical behavior is necessary to be improved by further experiments.

### Zero-Dimensional Alloying Materials

Alloying materials including Sb, Sn, Bi, Ge, as well as P, characterized by high capacity, have been studied for PIBs anode materials [84]. Volume expansion is one of their most obvious shortcomings, and the stress variation can be affected by small size structures. Therefore, it is urgent to design appropriate electrode materials and designing suitable nanostructures is a common modification method.

**Fig. 5:**
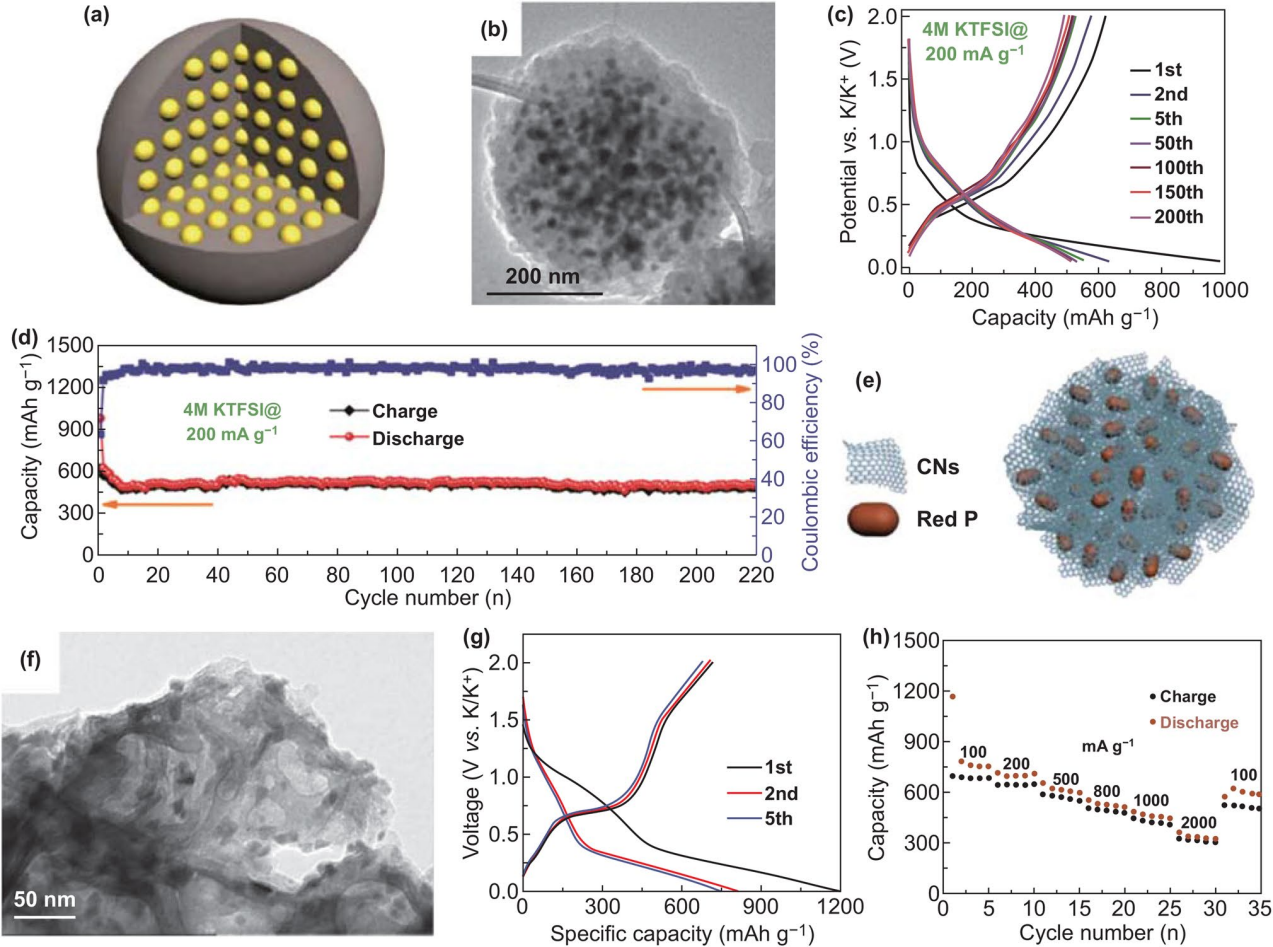
**a** Sb@CSN’s schematic diagram. **b** Single Sb@CSN sphere exhibited in TEM image. **c** Voltage profiles as well as **d** Related cycle capacities of Sb@CSN at 200 mA g^−1^. Reproduced with permission from Ref. [90]. Copyright 2019, Royal Society of Chemistry. **e** Schematic illustration of red P@CN composite. **f** TEM image of red P@CN composite. **g** Voltage curves of red P@CN composite for the selected cycles. **h** Rate capabilities of red P@CN composite in the range of 100–2000 mA g^−1^. Reproduced with permission from Ref. [122]. Copyright 2018, John Wiley and Sons

It was reported that nanoparticles have been widely fabricated as electrode materials to mitigate stress changes. As a typical example, engineering bulk materials to nanoparticles is beneficial to alleviate stress variation. Thus, the network of a carbon sphere enclosed Sb nanoparticles (Sb@CSN) as PIBs anode [90]. The schematic diagram of Sb@CSN and its TEM image are shown in Fig. 5a, b, which indicated the Sb nanoparticles were well encapsulated in the carbon sphere with uniform distribution. As for electrochemical performances, the Sb@CSN electrode presented high discharge capacity of 626 mAh g^−1^ at 200 mA g^−1^ after second cycle (Fig. 5c). In addition, the capacity of 504 mAh g^−1^ was obtained at 200 mA g^−1^ after 200 cycles (Fig. 5d). The Sb@CSN anode’s electrochemical behavior indicated the importance of small size nanoparticles and carbon sphere network, which could buffer volume expansion and promote the transportation of electrons. Similarly, Ge and co-workers [87] also constructed a composite composed of Sb nanoparticles and carbon materials. They encapsulated ultra-small Sb nanocrystals into CNFs for PIBs anode, which achieved 225 mAh g^−1^ at 1 A g^−1^ after 2000 cycles because ultra-small Sb nanocrystals and hollow nanochannels not only promoted K^+^ fast diffusion but also buffered strain variation. Compared to carbon spheres, CNFs may have better connectivity and can better make the electrode stable. Additionally, the nanostructural design also has an impact on the electrochemical performances of red P. So tailoring different sizes of red P into 3D carbon could effectively overcome the above-mentioned disadvantages (Fig. 5e) [122]. In this composite, the small size of red P and 3D carbon nanosheet framework could reduce the degree of volume expansion; on the other hand, it also could boost the fast electron transportation during charge and discharge process. In addition, red P without agglomeration was displayed, which indicated that red P was uniformly dispersed in the 3D carbon nanosheet frameworks (Fig. 5f). Benefit from the structural design, the red P@CN composite anode delivered a charge capacity of 715.2 mAh g^−1^ and a rate capacity of 323.7 mAh g^−1^ at 2000 mA g^−1^ (Fig. 5g, h). Based on nanoparticles with effective impact, different morphology of 0D alloying materials should be designed to enhance the electrochemical performances.

## One-Dimensional Nanomaterials for PIBs

1D nanomaterials are defined as high length-to-diameter aspect ratios, including nanotubes, nanorods, nanowires, nanofibers, and nanoribbons. Since CNTs were first discovered by Iijima [123], 1D nanomaterials with similar structures have been widely studied. The structural features of 1D nanomaterials not only include nanoscale and microscale joint effects, but also contain high aspect ratio and oriented growth direction [124]. Therefore, 1D nanomaterials are beneficial for fast transportation of electrons and ions and have an impact on tolerating the stress changes, which was considered as a class of most promising materials for high performances of energy storage systems [125]. Given these mentioned above, 1D nanomaterials can be utilized to boost the diffusion of electrons and ions as well as alleviate the stress variation as PIBs electrode materials. Significant progress has recently been achieved for anode materials with high performances by designing and fabricating 1D nanomaterials. Based on typical 1D nanomaterials, systematic discussion is carried out about the relationship between the related structures and their electrochemical performances. Furthermore, in order to comprehensively comprehend the electrochemical behavior of 1D nanomaterials, the initial C.E., rate performances, and cycle properties of recent reported 1D anode materials of PIBs are outlined in Table 2.

**Table 2:** Comparison of state-of-the-art performances of 1D anode materials in PIBs

Materials	Initial C.E. (%)	Rate capacity (mAh g^−1^) at the current density (mA g^−1^)	Cycle capacity (mAh g^−1^) at the current density (mA g^−1^) (cycle number)	References
Ti_3_C_2_	–	60 at 300	42 at 200 (500)	[52]
Cryptomelane-type MnO_2_/CNT hybrids	40.07	127.2 at 1000	226.5 at 100 (500)	[126]
C-NCNFs	37.8	84.7 at 5 C	103.4 at 2 C (500)	[53]
b-NCNTs	23.3	186 at 1000	204 at 500 (1000)	[54]
M-NCNTs	24.45	102 at 2000	102 at 2000 (500)	[55]
Highly N-doped CNFs	49	101 at 20,000	146 at 2000 (4000)	[81]
Cup-stacked NCNT mats	14.2	75 at 1000	236 at 20 (100)	[127]
Red P	68.26	71 at 3000	300 at 1000 (60)	[56]
Sb/HCT	70	211.5 at 5000	300.1 at 2000 (120)	[57]
3D amorphous carbon encapsulated CoS/NCNTs on CoS-coated CNFs	57.6	133.1 at 6400	≈130 at 3200 (600)	[128]
Carbon-encapsulated CoP nanoparticles embedded in CNTs supported on CNFs	53.2	~292 at 3200	247 at 800 (1000)	[129]
Sub-micro-carbon fiber@CNTs	–	108 at 5 C	more than 193 at 1 C (300)	[130]
Pyrrolic/pyridinic-N-doped necklace-like hollow carbon	–	204.8 at 2000	161.3 at 1000 (1600)	[131]
Bi-nanorod networks confined in N, S co-doped carbon matrix	65	289 at 6000	285 at 5000 (1000)	[132]
rGO/CNT hybrid papers	–	110 at 100	148 at 50 (200)	[133]
Porous Mn–Fe-Se composite adhered/inserted with interlaced CNTs	33.43	83 at 800	141 at 50 (70)	[134]
Hollow NCNFs anchored hierarchical FeP nanosheets	57	103 at 800	210 at 100 (1000)	[135]

### One-Dimensional Carbon Materials

**Fig. 6:**
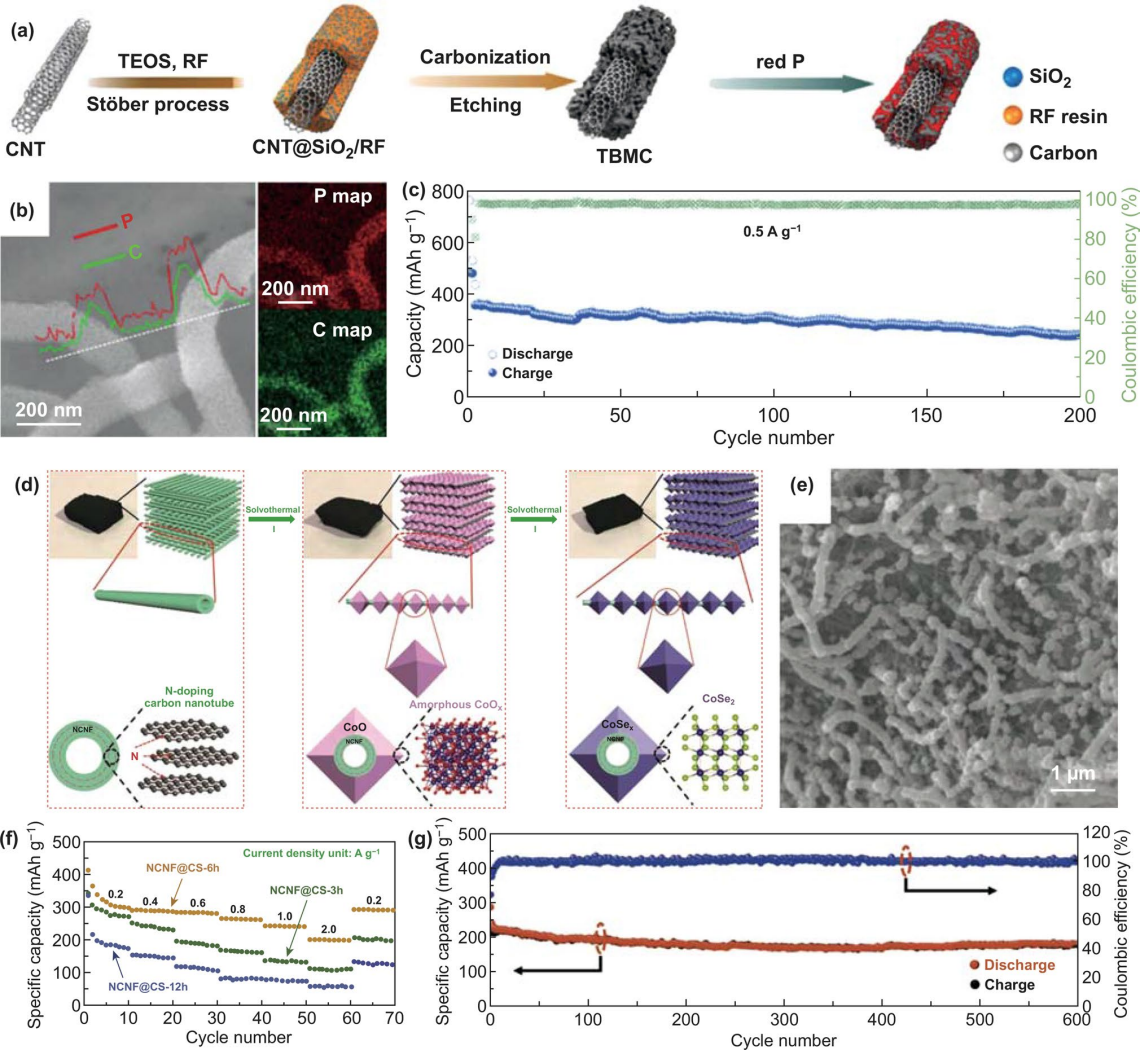
**a** Schematic diagram for P@TBMC composite’s fabrication process. **b** The corresponding EDS elemental mapping and line scanning of P@TBMC-2.4 displayed in SEM image. **c** Long-term cycle capacities at 0.5 A g^−1^ after initial two-cycle activation at 0.05 A g^−1^. Reproduced with permission from Ref. [50]. Copyright 2018, Elsevier. **d** Schematic diagram for NCNF@CS’s preparation process. **e** SEM image of NCNF@CS-6 h. **f** Rate capacities of the obtained three NCNF@CS samples. **g** Long-term cycling stability and C.E. of NCNF@CS-6 h at 2.0 A g^−1^ over 600 cycles. Reproduced with permission from Ref. [51]. Copyright 2018, John Wiley and Sons

CNTs can be considered as a representative example among 1D carbon materials. It is well established that CNTs with remarkable mechanical strength and excellent electrical properties have been applied in PIBs field [136–139]. So far, CNTs not only can be used directly or doped with other elements, but also can be composited with other materials to serve as electrode materials. As for composites, a CNT-backboned mesoporous carbon confining red P composite (P@TBMC) was formed as anode material for SIBs/PIBs (Fig. 6a) [50]. P encapsulated completely within the pores, and homogenous distributions in the composite were confirmed (Fig. 6b). In these composites, the main function of red P was to provide high K storage capacity. The multi-walled CNTs not only supported the structure as backbone, but also boosted the electrons transfer because of the high content of *sp*^2^ carbon. Therefore, the P@TBMC-2.4 (2.4 means the resorcinol mass (g) in the recipe) anode exhibited a depotassiation capacity of 244 mAh g^−1^ at 0.5 A g^−1^ after 200 cycles, which displayed stable cyclability (Fig. 6c).

Generally, heteroatom-doped carbon materials have better K storage properties than non-doped ones, so heteroatom-doped CNTs may perform better than ordinary CNTs. So a CoSe_2_ strung by CNTs with N-doping (NCNF@CS) was prepared by two-step hydrothermal means as a PIBs anode (Fig. 6d) [51]. Then, the CNTs made every octahedral CoSe_2_ particle arrange in sequence with zigzag void space among particles (Fig. 6e). Remarkably, the NCNF@CS-6 h (6 h means the reaction hours) anode reached the reversible capacity of 196 mAh g^−1^ at 2.0 A g^−1^ (Fig. 6f). Moreover, a capacity of 173 mAh g^−1^ at 2.0 A g^−1^ after over 600 cycles was exhibited (Fig. 6g). The electrochemical performances of NCNF@CS-6 h electrode were ascribed to the use of CNTs and octahedral CoSe_2_ particles arranging in sequence, where conductive N-doped CNTs not only hindered the agglomeration and anchored the active materials as backbone but also effectively promoted the electronic transfer. Based on aforementioned work, CNTs can enhance the electrochemical behaviors by boosting electronic and ionic diffusion and keeping structural integrity. Therefore, novel composites about CNTs deserve to research for PIBs anode materials in the further experiments.

### One-Dimensional MCs and MOs

**Fig. 7:**
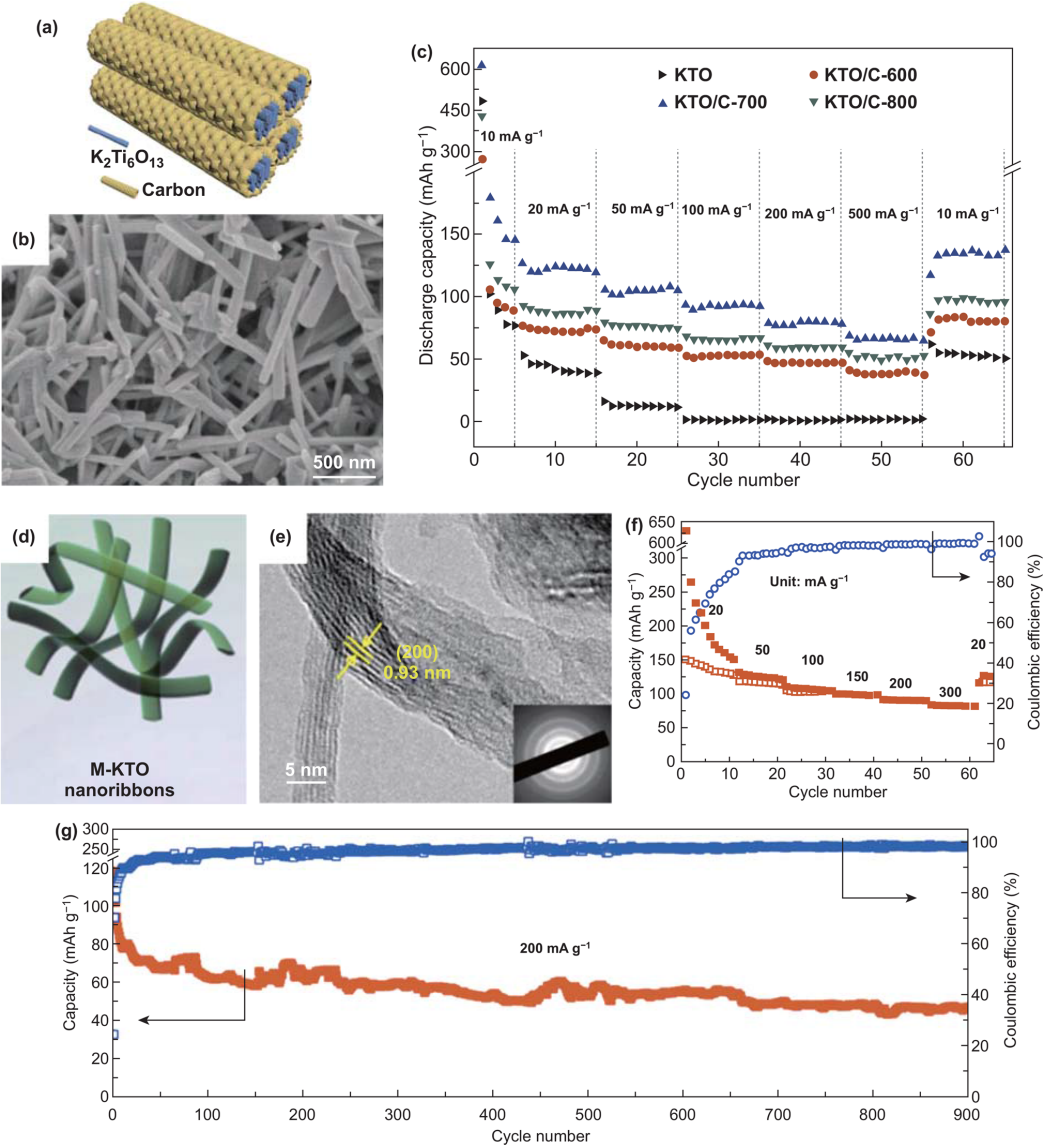
**a** Schematic diagram of KTO/C. **b** SEM image of KTO/C-700. **c** Rate capacities of the obtained hybrids electrode. Reproduced with permission from Ref. [140]. Copyright 2020, Royal Society of Chemistry. **d** M-KTO nanoribbons’ schematic illustration. **e** M-KTO’s HRTEM image with the corresponding SAED patterns (inset). **f** M-KTO’s rate performance. **g** Long cycle capacities as well as C.E. of the obtained sample at 200 mA g^−1^. Reproduced with permission from Ref. [92]. Copyright 2017, American Chemical Society

To date, 1D MCs and MOs mainly include various K-Ti–O anode materials with common formula K_2_Ti_n_O_2n+1_, which are advantageous for insertion/extraction of K-ion [140]. As for K_2_Ti_6_O_13_, its crystal structure with open 3D framework and tunnel can facilitate K-ion transfer. So K_2_Ti_6_O_13_ nanorods were synthesized as PIBs anode and the obtained electrode achieved stable long cycling performance, but its poor rate performance was exhibited [58]. Accordingly, carbon coating may be a practical way to further improve the rate capacities. Therefore, oriented nanorod-like K_2_Ti_6_O_13_ bunches with a thin carbon layer (KTO/C) were fabricated as PIBs electrode materials (Fig. 7a) [76]. The size of nanorods of KTO/C-700 (700 means the heat treatment temperature of 700 °C) was well maintained compared with that of bare KTO (Fig. 7b). As for rate capacities, the KTO/C-700 electrode delivered 65.1 mAh g^−1^ at 500 mA g^−1^ (Fig. 7c). The electrochemical performances of the KTO/C-700 electrode were due to K-ion diffusion boosted by crystal orientations of KTO and electron conductivity improved by carbon layer. Besides, in 2016, K_2_Ti_4_O_9_ was fabricated as PIBs anode materials for the first time, but delivered low capacities and poor cycling performance [141]. It is necessary to control the morphology of K_2_Ti_4_O_9_ well so as to improve the electrochemical behavior. Therefore, K_2_Ti_4_O_9_ nanoribbons derived from MXene have been studied for PIBs anode materials due to unique structural features. Wu’s group [92] reported that ultrathin nanoribbons of potassium titanate (M-KTO, K_2_Ti_4_O_9_) were synthesized as anode materials for PIBs. The morphology of nanoribbons and the interlayer space with 0.93 nm were exhibited, which benefited K-ion insertion/extraction (Fig. 7d, e). As for electrochemical performances, the rate capacity of 81 mAh g^−1^ was attained at 300 mA g^−1^ as well as the retention of 51% (of the second charge capacity) was exhibited at 200 mA g^−1^ after over 900 cycles (Fig. 7f, g). The M-KTO electrode’s electrochemical performances were ascribed to suitable interlayer spacing, narrow widths, ultrathin thickness, as well as open macroporous architectures. The convincing example verified that nanoribbons are beneficial to enhance electrochemical behavior due to well-designed structures. However, because only single material is used, the K storage capacity is not high enough, and it may need to be compounded with other materials in the subsequent research to further improve the electrochemical performances.

### One-Dimensional Alloying Materials

Up to now, few studies about 1D alloying materials have involved in nanotubes, nanowires, nanofibers, and nanoribbons in the PIBs fields. Therefore, nanorod-like alloying materials with shorter ionic transfer path will be introduced by typical examples [57, 59, 91, 132, 142].

**Fig. 8:**
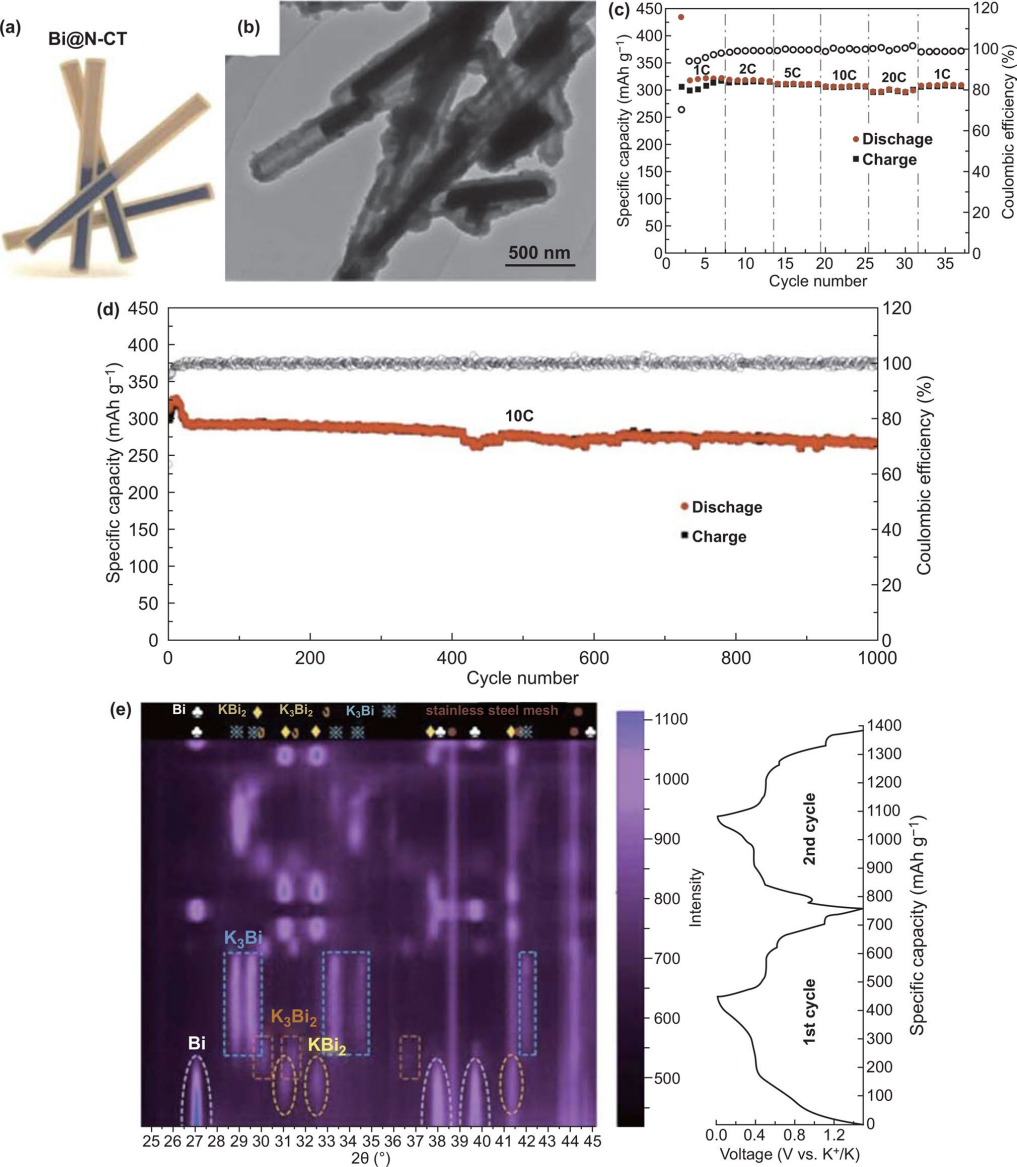
**a** Bi@N–CT’s schematic diagram. **b** Bi@N–CT’s TEM image. **c** Rate performance of the obtained electrode at different C-rates (1 C is 385 mAh g^−1^). **d** Long-term cycling performance of Bi@N–CT at 10 C. **e** Contour plot of the obtained electrode’s in situ XRD results in the course of discharge/charge process about the first two cycles with discharge/charge curves. Reproduced with permission from Ref. [91]. Copyright 2020, Royal Society of Chemistry

Nanorod-like Bi has been composited with carbon materials as anode materials for PIBs. For instance, Bi nanorods coated with mesoporous carbon were fabricated [142]. The unique structure with the core of Bi nanorods and outside carbon mesoporous shell had an effect on boosting the electronic and ionic diffusion as well as buffering the volumetric variation. Thus, the storage capacity reached 425 mAh g^−1^ at 0.2 A g^−1^. Compared with mesoporous carbon coatings, CNTs may encapsulate and confine Bi nanorods better due to their hollow tubular structures and good mechanical properties. Li et al. [91] synthesized Bi nanorods confined by hollow N-doped CNTs (Bi@N–CT) as anode materials of PIBs. The Bi nanorod structure was encapsulated in hollow structure with the carbon coating layer thickness of about 40 nm (Fig. 8a, b). In the electrochemical test, the capacity was 297 mAh g^−1^ at 20 C (Fig. 8c). Additionally, the obtained electrode reached the capacity of 266 mAh g^−1^ at 10 C after over 1000 cycles (Fig. 8d). These electrochemical performances were largely ascribed to the nanostructural design as well as the perfect coordination of Bi nanorods and CNTs. In addition, the storage mechanism could be understood well by in situ XRD, which indicated the high reversibility of structure (Fig. 8e). Coincidentally, Bi nanorods were encapsulated in N-doped CNTs (Bi@C nanorods) for PIBs anode [59]. The N-doped CNTs were originated from the carbonization of polydopamine, different from the above example using polypyrrole. The morphology of Bi nanorods, hollow structural robustness, and carbon coating layer conductive network were beneficial for the obtained anode to deliver the capacity of 179.1 mAh g^−1^ at 0.5 A g^−1^ after over 300 cycles. Therefore, Bi nanorods compounded with hollow structure carbon materials are advantageous to restrict volume changes and keep long cycle stability.

## Two-Dimensional Nanomaterials for PIBs

2D nanomaterials have attracted increasing attentions since the discovery of graphene in 2004, and they have been extensively studied for electrochemical energy storage because of unique structural features as well as physicochemical properties [143–151]. On the one hand, the large surface area of 2D nanomaterials is conducive to ionic adsorption, which is beneficial to improve capacitance. On the other hand, 2D nanomaterials with high conductivity and tunable interlayer spacing can boost electronic transfer and benefit ionic intercalation, respectively [152]. Based on these advantages, 2D materials have become favored materials for researchers. Therefore, the corresponding 2D materials will be introduced typically. And then, the initial C.E., rate performances, and cycle properties of recent reported 2D anode materials of PIBs are summarized in Table 3.

**Table 3:** Comparison of the state-of-the-art performances of 2D anode materials in PIBs

Materials	Initial C.E. (%)	Rate capacity (mAh g^−1^) at the current density (mA g^−1^)	Cycle capacity (mAh g^−1^) at the current density (mA g^−1^) (cycle number)	References
Nanocrystalline SnS_2_ coated onto rGO	–	120 at 2000	280 at 25 (25)	[153]
Ti_3_CNTz	28.4	32 at 500	75 at 20 (100)	[60]
MoS_2_@rGO	–	178 at 500	381 at 100 (100)	[61]
MoS_2_@SnO_2_@C	73	86 at 800	250 at 100 (20)	[62]
MoS_2_/C	–	164 at 2000	180 at 500 (240)	[154]
Envelope-like N-doped carbon nanosheets	≈20	168 at 2000	151 at 1000 (1000)	[155]
VSe_2_	69.1	172 at 2000	≈87.3% of the capacity retention at 2000 (500)	[63]
FSCC	62.7	139 at 1000	226 at 100 (100)	[156]
SnS_2_/graphene	40.5	290 at 2000	559 at 100 (50)	[157]
CuO nanoplates	50.8	163 at 2000	206 at 1000 (100)	[158]
SnS_2_/rGO	51.2	247 at 1000	205 at 1000 (300)	[64]
Amorphous carbon/graphitic carbon nanoplates	15.7	120 at 5000	192 at 1000 (5200)	[159]
SnS_2_@C@rGO	53.0	287.8 at 500	170.9 at 500 (500)	[160]
HeTiO_2_eC micro-tubes	49.1	97.3 at 2000	132.8 at 500 (1200)	[161]
MnCO_3_ nanorods@rGO	–	98 at 2000	701 at 200 (500)	[162]
N and P co-doped vertical graphene/carbon cloth	53.47	156.1 at 2000	142.4 at 1000 (1000)	[163]
rGO@p-FeS_2_@C composite	–	298 at 2000	322 at 1000 (30)	[164]
SnP_0.94_ nanoplates/graphene oxide composite	42	57 at 1000	106 at 200 (100)	[165]
Activated crumbled graphene	≈39	210 at 2000	245 at 500 (2800)	[166]

### Two-Dimensional Carbon Materials

**Fig. 9:**
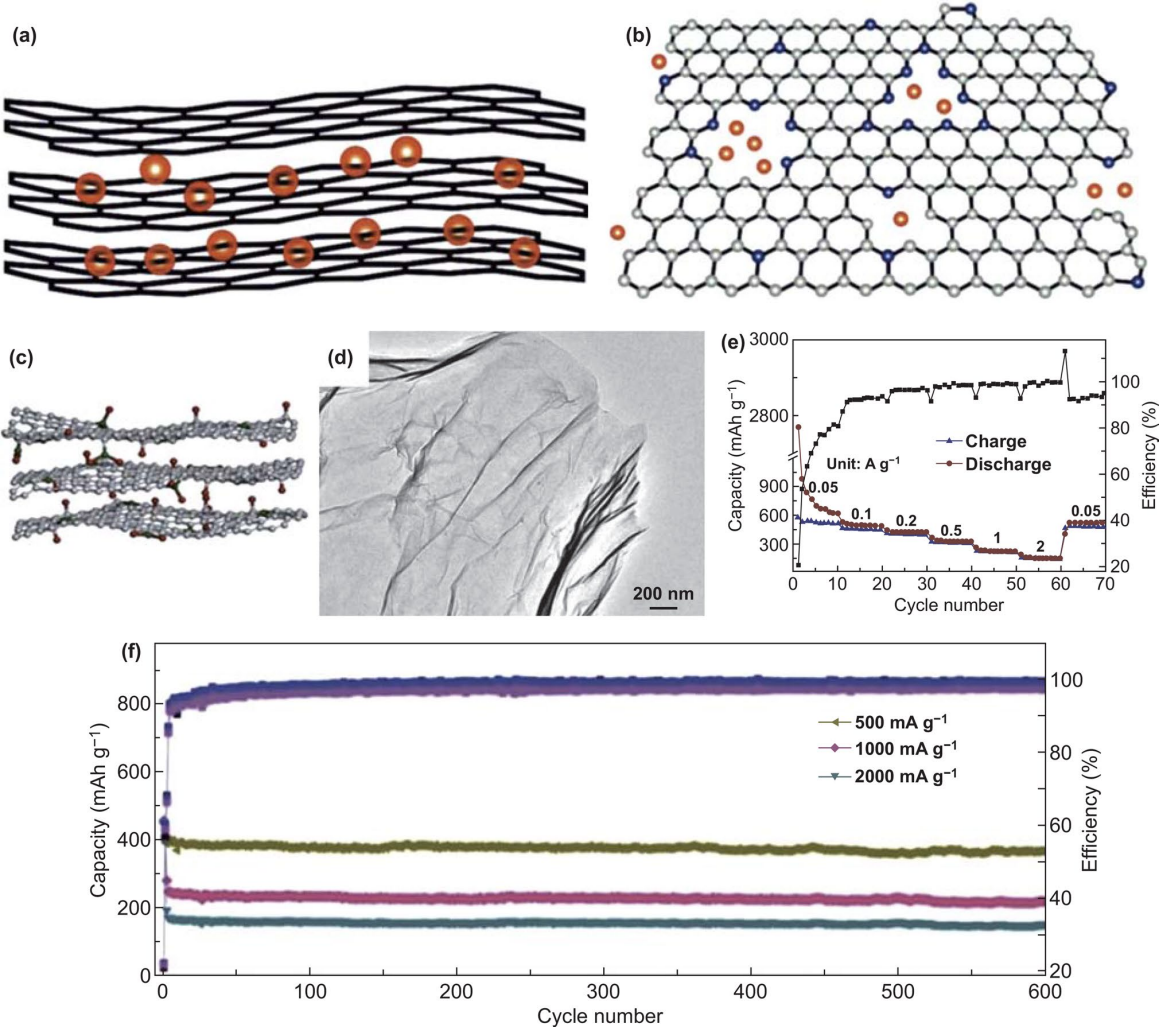
Schematic diagram of K^+^ trapped **a** in the surface and **b** in the defect sites. Reproduced with permission from Ref. [65]. Copyright 2018, Elsevier. **c** Schematic diagram and **d** TEM image of PODG. **e** The rate capability of the PODG electrode. **f** Cycle capacities of the obtained anode under three different current densities. Reproduced with permission from Ref. [66]. Copyright 2017, Royal Society of Chemistry

As a typical example of 2D carbon materials, graphene has been used for PIBs because of its high surface area, extraordinary mechanical strength, as well as high electrical conductivity [167–169]. In 2015, it was the first time that the K-ion intercalation of rGO film was studied by Luo and co-workers [170]. Although the reversible capacity was 222 mAh g^−1^, rGO with poor rate capability was due to its inferior electronic conductivity. Therefore, graphene need to be modified and modifying its structure may be one of the effective strategies to improve its electrochemical performances. Firstly, single heteroatom doping has been used to regulate the structure of graphene. Few-layered graphene with N-doping was reported as PIBs anode materials, which realized the charge capacity of over 350 mAh g^−1^ at 50 mA g^−1^ and the cycle capacity of more than 210 mAh g^−1^ at 100 mA g^−1^ after 100 cycles because the obtained electrode could provide abundant sites for ion storage and boost ionic transfer [171]. Based on an improved method, similar few-layer N-doped graphene (FLNG) was prepared as electrode materials in PIBs [65]. Its storage mechanism was made up of two parts including trapping K-ion on the surface and into the defect sites (Fig. 9a, b). The FLNG could supply more K-ion storage active sites and enhance electronic as well as ionic diffusion because of few-layer structure, N-doping impact and high surface area, so the long cycle capacity of 150 mAh g^−1^ was realized at 500 mA g^−1^ after 500 cycles. Besides, in order to regulate the structure of graphene better, co-doping with two different heteroatoms has been utilized. Ma et al. [66] fabricated graphene with P and O co-doping (PODG) as PIBs electrode materials (Fig. 9c). The ultrathin film of PODG with only a few layers was displayed in the TEM image (Fig. 9d). As for PODG, graphene and elements with P and O not only boosted K-ion diffusion by the expanding interlayer spacing, but also promoted K-ion adsorption because of large surface area as well as sufficient defects. Therefore, the PODG electrode delivered 165 mAh g^−1^ at 2000 mA g^−1^ (Fig. 9e). Moreover, PODG electrode delivered about 385, 235, and 160 mAh g^−1^ at 500, 1000, as well as 2000 mA g^−1^ after 600 cycles, respectively (Fig. 9f). Accordingly, expanding interlayer spacing and abundant active sites can be achieved by dual doping, so Luan and co-workers [172] further synthesized the multilayer graphene with N and P dual-doping (NPG) in PIBs electrode materials field. As for its electrochemical behavior, the rate capacity of 194 mAh g^−1^ was achieved at 1000 mA g^−1^. In addition, the cycle capacity of 242 mAh g^−1^ was obtained at 500 mA g^−1^ after 500 cycles. These electrochemical performances were mainly due to the NPG with sufficient active sites for ion storage, expanding interlayer spacing, and enhanced electrochemical conductivity. Given the aforementioned examples, graphene can improve electrochemical performances of PIBs anode by heteroatom doping (e.g., N, P, and O). However, the obstructions of graphene in practical applications are attributed to low initial coulomb efficiency, potential plateau lack, and the large voltage hysteresis [173]. Therefore, some strategies should be undertaken in the further experiments.

### Two-Dimensional MCs and MOs

**Fig. 10:**
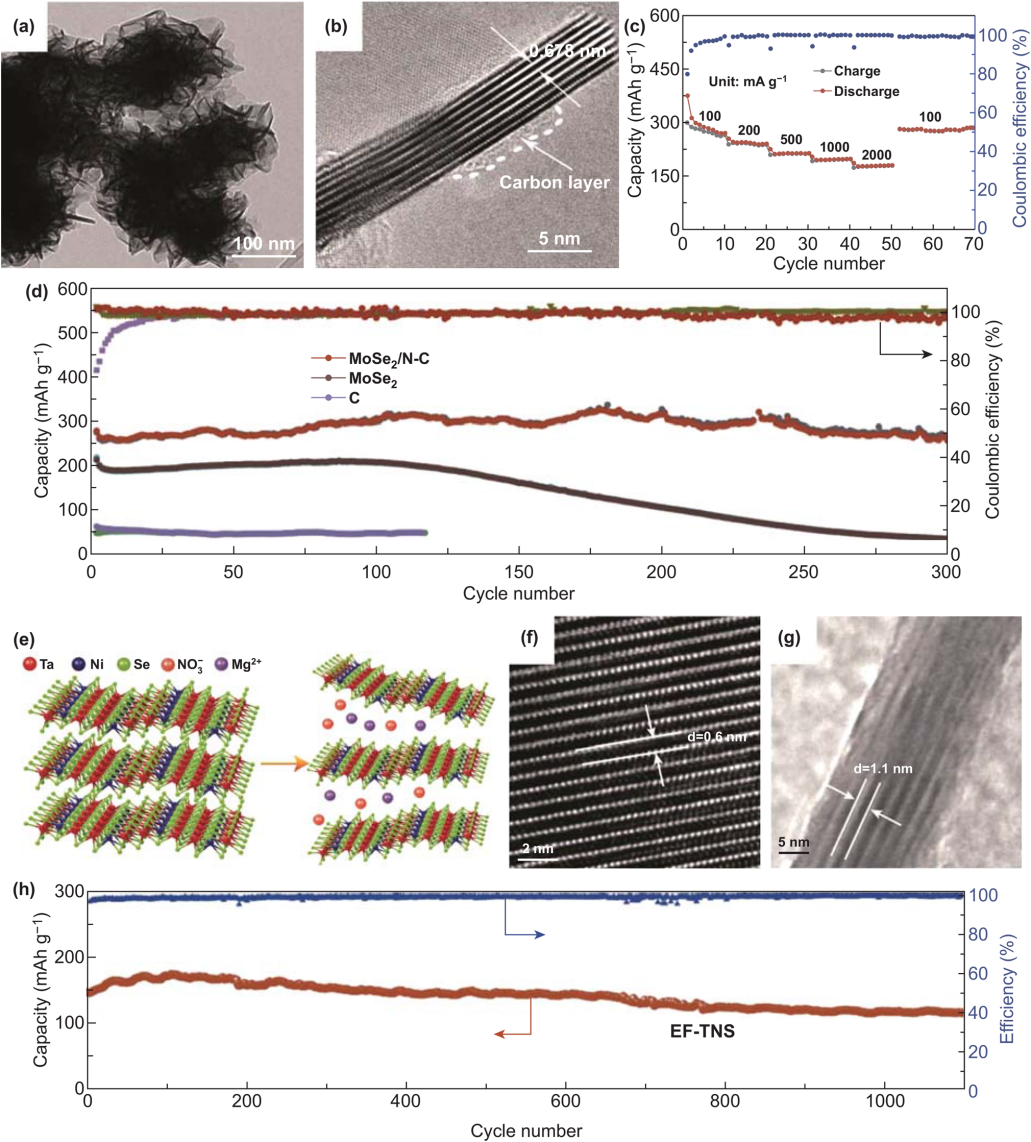
**a** TEM image of MoSe_2_/N–C. **b** HRTEM image of MoSe_2_/N-C. **c** Rate capability of MoSe_2_/N-C from 100 to 2000 mA g^−1^. **d** Long Cycle capacities of MoSe_2_/N-C, MoSe_2_, as well as C at 100 mA g^−1^. Reproduced with permission from Ref. [67]. Copyright 2018, John Wiley and Sons. **e** Schematic diagram of expanding process of EF-TNS. **f** HRTEM image of pristine Ta_2_NiSe_5_. **g** HRTEM image of intercalated Ta_2_NiSe_5_. **h** Stable cycle capacities of EF-TNS anode at 500 mA g^−1^. Reproduced with permission from Ref. [68]. Copyright 2019, John Wiley and Sons

Among 2D MCs and MOs, 2D transition metal chalcogenides (2D TMCs), including MoS_2_, MoSe_2_, VS_2_, VSe_2_, V_5_S_8_, etc., have been studied in PIBs anode materials for the reason that they have sufficient active sites, short ionic transfer pathways, and low intercalation barriers [174]. Taking MoSe_2_ as an example, it possesses sandwich-like lamellar structure, but low intrinsic conductivity, compounding with carbon materials, could be considered to enhance its electrochemical performances. So MoSe_2_/N-doped carbon (MoSe_2_/N–C) composite was synthesized as anode materials in PIBs [67]. Spherical structure was composed of a great number of nanosheets, and the interplanar spacing with 0.678 nm was beneficial to K^+^ insertion/extraction (Fig. 10a, b). In addition, the coated carbon layer encapsulating the MoSe_2_ nanosheets effectively improved conductivity and obstructed the aggregation of the nanosheets. As for electrochemical performances, the MoSe_2_/N-C electrode remained 178 mAh g^−1^ at 2000 mA g^−1^ as well as maintained 258.2 mAh g^−1^ after 300 cycles at 100 mA g^−1^ (Fig. 10c, d). Besides, quite a few binary 2D TMCs have also been reported to improve the electrochemical performances of PIBs anode, but a few studies about ternary 2D TMCs have been published. Gradually, ternary 2D TMCs have aroused increasing interesting in PIBs anode due to their unique structure and properties [68, 175, 176]. Then, expanded few-layered ternary Ta_2_NiSe_5_ (EF-TNS) flakes were reported [68]. The Ta_2_NiSe_5_ with expanded interlayer was realized by Mg_2_^+^/NO_3_^−^ ion assisted intercalation (Fig. 10e). First of all, ternary Ta_2_NiSe_5_ was beneficial to increase the capacity. Secondly, the obtained EF-TNS had a few-layered structure, which was in favor of buffering the volume variation. Thirdly, the interlayer distance of EF-TNS was expanded from 0.6 to 1.1 nm after intercalation, which facilitated K-ion diffusion and benefited for K^+^ ions intercalating along zigzag pathways (Fig. 10f, g). These three advantages made it have good K storage performances and become promising anode materials. As a consequence, the EF-TNS electrode delivered the capacity of 116 mAh g^−1^ at 500 mA g^−1^ after 1100 cycles (Fig. 10h).

In order to further search for appropriate anode materials, 2D metal-based oxides have been studied in PIBs [177]. Sb_2_O_3_ flakes anchored onto rGO were synthesized by Li and co-workers [178], which delivered the long cycle capacity of 201 mAh g^−1^ at 500 mA g^−1^ after 3300 cycles. As for the reason of the remarkable electrochemical behavior, it was found that the Sb_2_O_3_ flakes and rGO could not only make electrode and electrolyte close, but also enhance conductivity and buffer the volume expansion. In addition, further strategies should be taken so as to relieve the stress variation of Sb_2_O_3_ better, such as designing rational structures and synthesizing bimetallic compounds; especially, bimetallic compounds with the better electrochemical behavior are ascribed to the synergistic effects, more active sites, and interfacial effects, compared with the single-metal counterparts [179–182]. Accordingly, Wang et al. [183] fabricated Sb_2_MoO_6_ nanoplates composited with rGO as anode materials of PIBs. In these composites, with the help of Mo element, the conductivity could be improved and the volume changes of Sb could be relieved in the course of charge and discharge process. Therefore, the electrochemical performances would be enhanced and the capacity of 247 mAh g^−1^ was delivered at 500 mA g^−1^ after 100 cycles.

### Two-Dimensional Alloying Materials

**Fig. 11:**
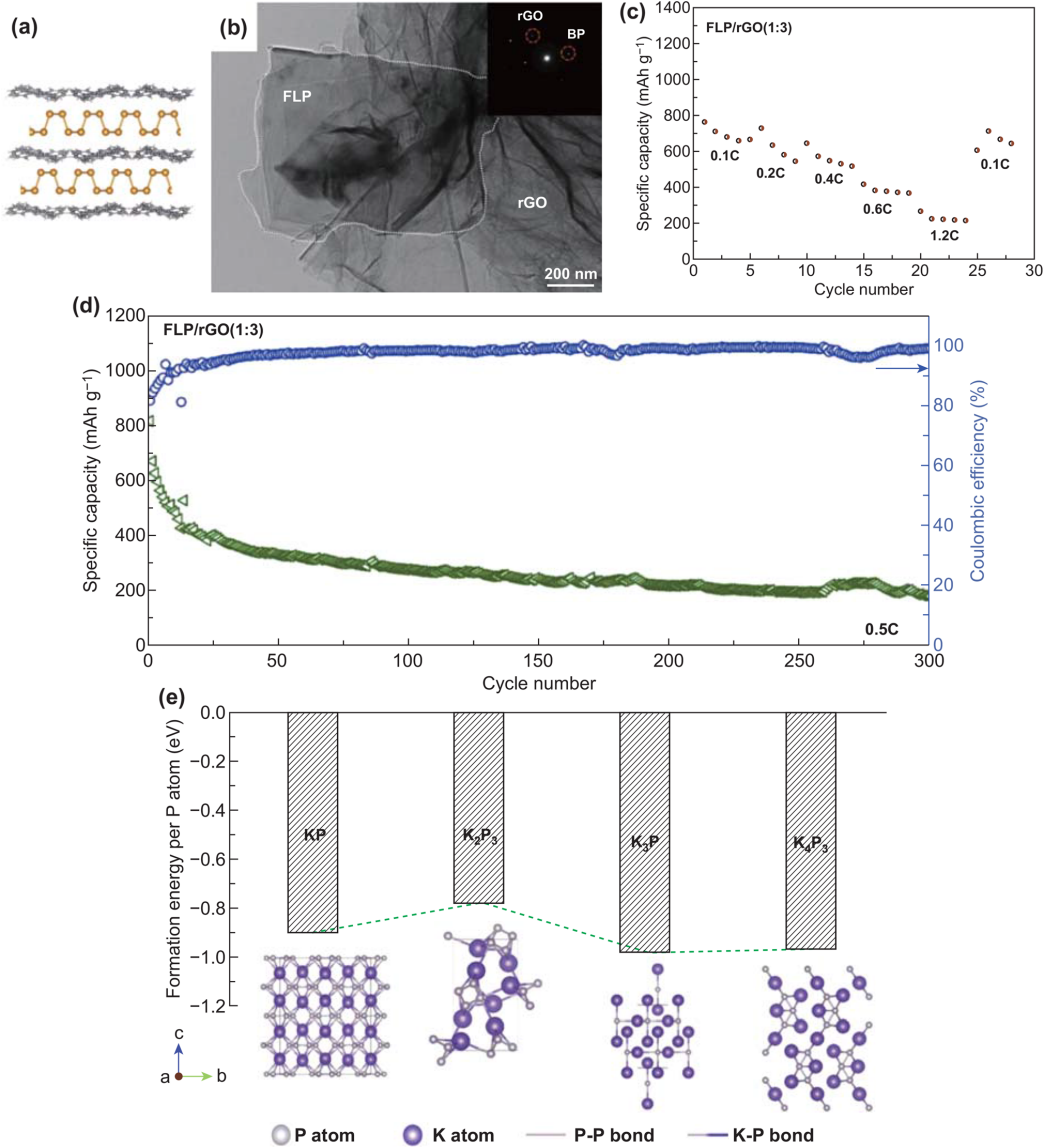
**a** Schematic diagram and **b** TEM image (inset shows SAED pattern) of FLP/rGO. **c** Rate capability of FLP/rGO (1:3) at various C-rates between ~ 0.1 and ~ 1.2 C. **d** Cycling performance of FLP/rGO (1:3) at ~ 0.5 C. **e** various alloys’ formation energies calculated by DFT. Reproduced with permission from Ref. [69]. Copyright 2019, American Chemical Society

Phosphorene can be regarded as a typical 2D alloying materials in PIBs. In 2014, monolayer and few-layer phosphorene were exfoliated from black phosphorus by scotch-tape-based micro-cleavage method [184, 185]. However, monolayer phosphorene could not be utilized directly as electrode materials, which was ascribed to be easily oxidized when it exposed to air [186, 187]. Therefore, He et al. [188] indirectly verified that the monolayer phosphorene was beneficial to improve performances of GeSe electrode by first-principles calculation. Moreover, phosphorene with sulfur doping could be considered as the PIBs anode materials by using ab initio density functional theory [189]. Furthermore, few-layer phosphorene has started to be directly utilized for PIBs because of high carrier mobility and superior mechanical flexibility [190]. And then, Nikhil Koratkar’s group [69] fabricated few-layer phosphorene composited with rGO (FLP/rGO) as anode materials (Fig. 11a). The TEM image of FLP/rGO indicated FLP encapsulated with rGO and the existing evidence of both FLP and rGO was provided by the SAED pattern (Fig. 11b). In these composites, FLP and rGO were beneficial for enhancing electrical conductivity as well as buffering volumetric variation in the course of alloying reaction. The FLP/rGO (1:3) electrode delivered different capacities at different current density, especially ~ 400 and ~ 230 mAh g^−1^ at ~ 0.6 C as well as ~ 1.2 C, respectively. As for cycle performance, FLP/rGO (1:3) delivered the capacity of ~ 230 mAh g^−1^ at ~ 0.5 C after 300 cycles (Fig. 11c, d). Furthermore, in terms of alloying mechanism, the author confirmed that K_4_P_3_ bringing about high capacity was the type of alloy and used DFT calculation to give a further understanding about the formation of K_4_P_3_ (Fig. 11e). Further experiments can focus on phosphorene compounded with other materials or doped with heteroatom to enhance the electrochemical performances.

## Three-Dimensional Nanomaterials for PIBs

3D nanomaterials are essential to the application of batteries, due to unique features such as high specific areas, interlinked porous channels, high conductivity and outstanding structural mechanical stability [191, 192]. 3D nanomaterials could overcome the insufficiency of 1D and 2D nanomaterials caused by evident aggregation [193]. In addition, some 3D nanomaterials can be directly used as free-standing electrodes, simplifying the preparation process of battery. To date, 3D nanomaterials in PIBs electrode materials field mainly include 3D carbon nanomaterials, 3D MCs and MOs, and 3D alloying materials [194–197]. At the following section, the relationship between these materials and their electrochemical performances will be discussed systematically. Meanwhile, the initial C.E., rate performances, and cycle properties of recent reported 3D anode materials of PIBs are summarized in Table 4.

**Table 4:** Comparison of the state-of-the-art performances of 3D anode materials in PIBs

Materials	Initial C.E. (%)	Rate capacity (mAh g^−1^) at the current density (mA g^−1^)	Cycle capacity (mAh g^−1^) at the current density (mA g^−1^) (cycle number)	References
N- and O-rich CNF	35	70 at 10 C	160 at 1 C (300)	[198]
Cobalt(II) terephthalate-based layered MOF	60.65	131 at 1000	188 at 1000 (600)	[199]
Tire-derived carbons	37.1	60 at 2 C	155 at C/2 (200)	[200]
Hard wood-based hard carbon	56	135 at 100	–	[201]
AC	–	30 at 1000	100.3 at 200 (100)	[74]
Porous CNF paper	24.1	140 at 5000	211 at 200 (1200)	[202]
S/O co-doped porous hard carbon microspheres	61.7	158 at 1000	108.4 at 1000 (2000 cycles)	[203]
Co_3_[Co(CN)_6_]_2_	45.5	112 at 2000	297.5 at 100 (200)	[75]
Skimmed cotton-derived hard carbon	73	165.2 at 4000	240 at 200 (150)	[204]
HPCS	45.3	150 at 500	276.4 at 500 (100)	[76]
SnO_2_-graphene-CNFs	44.13	114.81 at 1000	202.06 at 1000 (100)	[205]
MoS_2_/N-Doped C	–	131 at 2000	151 at 5000 (1000)	[206]
Hierarchically N-doped porous carbon	43.1	185 at 10,000	144.4 at 5000 (1000)	[89]
HNTO/CS	–	50 at 1000	88.9 at 1000 (1555)	[93]
High pyridine NPC	–	186.2 at 2000	231.6 at 5000 (2000)	[207]
Zero-strain potassium fluoromanganate hollow nanocubes	65	78 at 1000	110 at 4000 (10,000)	[208]
N-doped hierarchically porous carbon	30.28	193.1 at 500	121.3 at 5000 (1000)	[209]
KTi_2_(PO_4_)_3_@C nanocomposites	35.3	131.1 at 1000	69.7 at 1000 (1000)	[210]
NOHPHC	25	118 at 3000	230.6 at 500 (100)	[77]
Sn_4_P_3_ in N-doped carbon fibers	64.17	169.6 at 2000	160.7 at 5000 after 1000	[211]
Bi@3DGFs	51.1	113 at 10,000	164 at 1000 (400)	[212]
N-doped biomorphic carbon	55.1	102.6 at 2000	119.9 at 1000 (1000)	[213]
Nanosheets-assembled CuSe Crystal Pillar	92.4	280 at 5000	337 at 100 (40)	[214]
rGO aerogel	44	92 at 6.7 C	125 at 1.6 C (500)	[78]
KVPO_4_F	–	65 at 2000	133 at 100 (100)	[215]
MXene@Sb	57.29	270.81 at 500	capacity retention of 79.1435% at 500 (500 cycles)	[216]
Red P@N-PHCNFs	–	342 at 5000	282 at 5000 (800 cycles)	[217]
NOHPHC	–	110 at 1000	80 at 2000 (3000)	[218]
Yolk shell FeP@C nanoboxes	47	37 at 2000	205 at 1000 (300)	[219]
Multicore shell Bi@N-doped carbon nanospheres	43	152 at 100,000	203 at 10,000 (1000)	[220]
Graphitic nanocarbons	–	56.6 at 5000	189 at 200 (200)	[79]
HHC	46.88	42 at 3200	67.6 at 500 (100)	[80]
N-doped soft carbon frameworks built of well-interconnected nanocapsules	30.9	151 at 5000	165 at 1000 (500)	[221]

### Three-Dimensional Carbon Materials

**Fig. 12:**
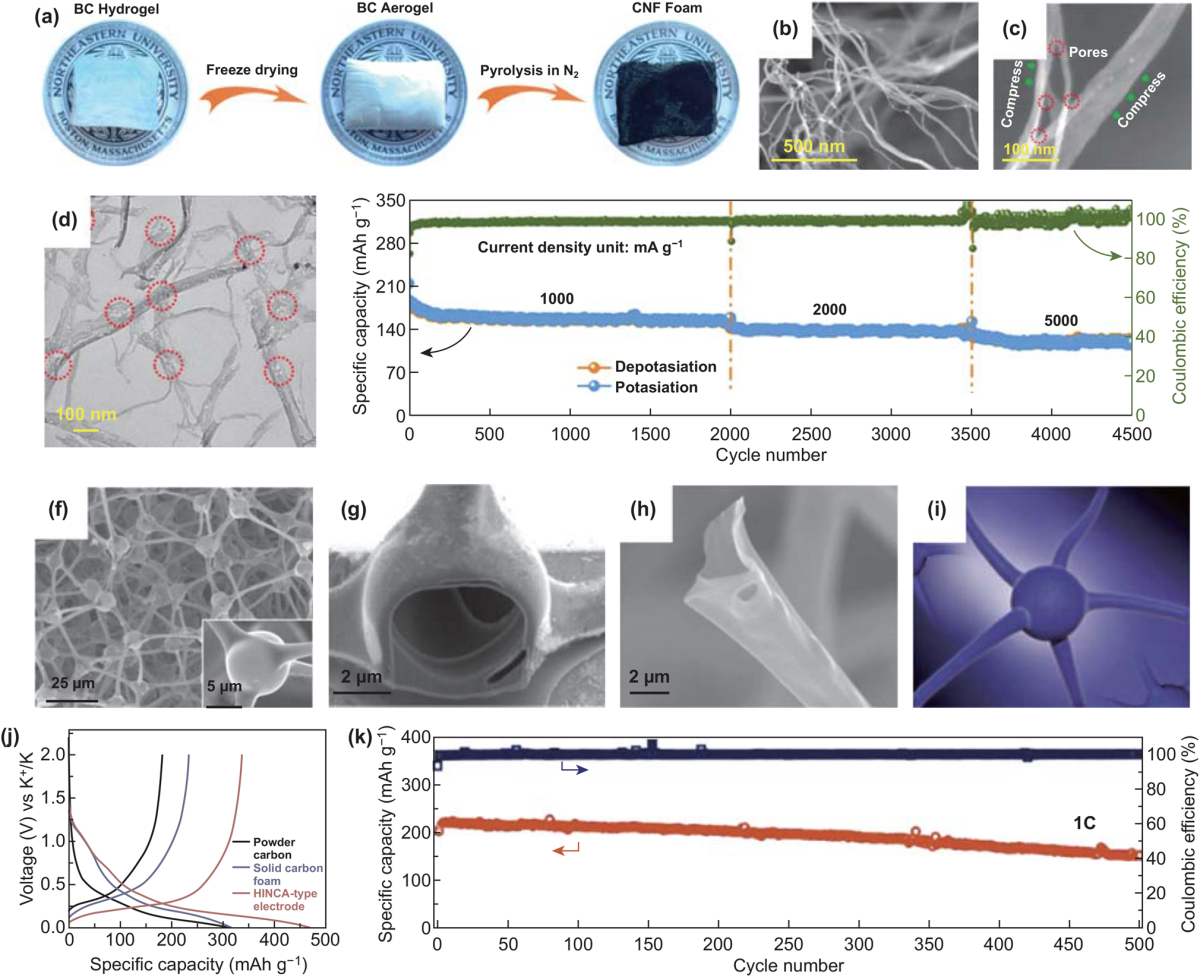
**a** Schematic diagram of preparation of CNFF. SEM images of **b** CNFF and **c** CNFs. **d** TEM image of CNFF. **e** Long-term cycling performance of CNFF electrode at three current densities. The battery rested for 10 days before using different current density. Reproduced with permission from Ref. [225]. Copyright 2018, American Chemical Society. **f** SEM image of HINCA and its tetrapod center (inset). SEM images of **g** the tetrapod-joint cross section and **h** a tetrapod-unit broken arm. **i** Neuron structural Cartoon. **j** Charge/discharge curves of the 1^st^ cycle at 0.1 C (28 mA g^−1^). k). Long cycle performance of the HINCA-type product at 1 C. Reproduced with permission from Ref. [226]. Copyright 2018, American Chemical Society

3D carbon materials mainly include 3D interconnected structures, which can promote electronic and ionic transport as well as improve mechanical stability [222–224]. Among 3D interconnected networks, 3D carbon nanofiber frameworks are typical materials and were used in PIBs anode materials field in 2018. Li et al. [225] reported bacterial-derived and compressible carbon nanofiber foam (CNFF) with hierarchical pores as electrode materials in PIBs (Fig. 12a). The diameters of fibers were between 10 and 30 nm shown in the SEM image (Fig. 12b). According to the authors’ report, two kinds of pores were found in this CNFF. The first was numerous nanopores originating from nanofibers’ surface; the other was hierarchical pores between fiber and fiber. Those pores could benefit K-ions absorption in the course of charge and discharge process (Fig. 12c, d). In the electrochemical test, the CNFF electrode achieved a reversible capacity of 158 mAh g^−1^ at 1000 mA g^−1^ after 2000 cycles. Furthermore, after a period of time, the same battery could also run 1500 and 1000 cycles at 2000 and 5000 mA g^−1^, respectively (Fig. 12e), showing superior cycle stability. The splendid electrochemical behavior of the CNFF anode was ascribed to the hierarchical pores of the structure, the 3D carbon foam, and the quasi-amorphous carbon, which not only enhanced ionic adsorption and diffusion but also relieved the volume variation. Additionally, 3D porous structures have also been used in PIBs. Bin et al. [226] fabricated interconnected carbon architecture with hollow structure and neuron-like morphology (HINCA) as anode materials. As for HINCA, interconnected tetrapod backbones with tubular structure and hollow structural spherical joint were exhibited and better comprehend by cartoon (Fig. 12f–i). The aforementioned structural characteristics could facilitate electronic conductivity and possess flexible mechanical robustness. Then, the HINCA-type electrode delivered the capacity of 340 mAh g^−1^ as well as showed only slight capacity decay of ~ 0.05% per cycle over 500 cycles at 1 C (Fig. 12j, k). The HINCA-type electrode’s electrochemical performances were due to carbon structure with hollow feature, which kept the stability of structure during potassiation/depotassiation process and promoted the ions and electrons transport. With similar structures, hollow neuronal carbon skeleton (HNCS) was fabricated, which exhibited interlinked hollow architecture with high content of pyridinic N [227]. The interlinked hollow framework and pyridinic N could enhance ionic transfer and adsorption as well as tolerate the stress variation to improve corresponding performances. As for electrochemical performances, the electrode delivered the rate capacity of 110 mAh g^−1^ at 1000 mA g^−1^ and 134 mAh g^−1^ at 0.5 A g^−1^ after 500 cycles. Overall, 3D carbon materials are beneficial to boost ionic and electronic transport and keep high mechanical robustness, resulting in improved electrochemical performances.

### Three-Dimensional MCs and MOs

**Fig. 13:**
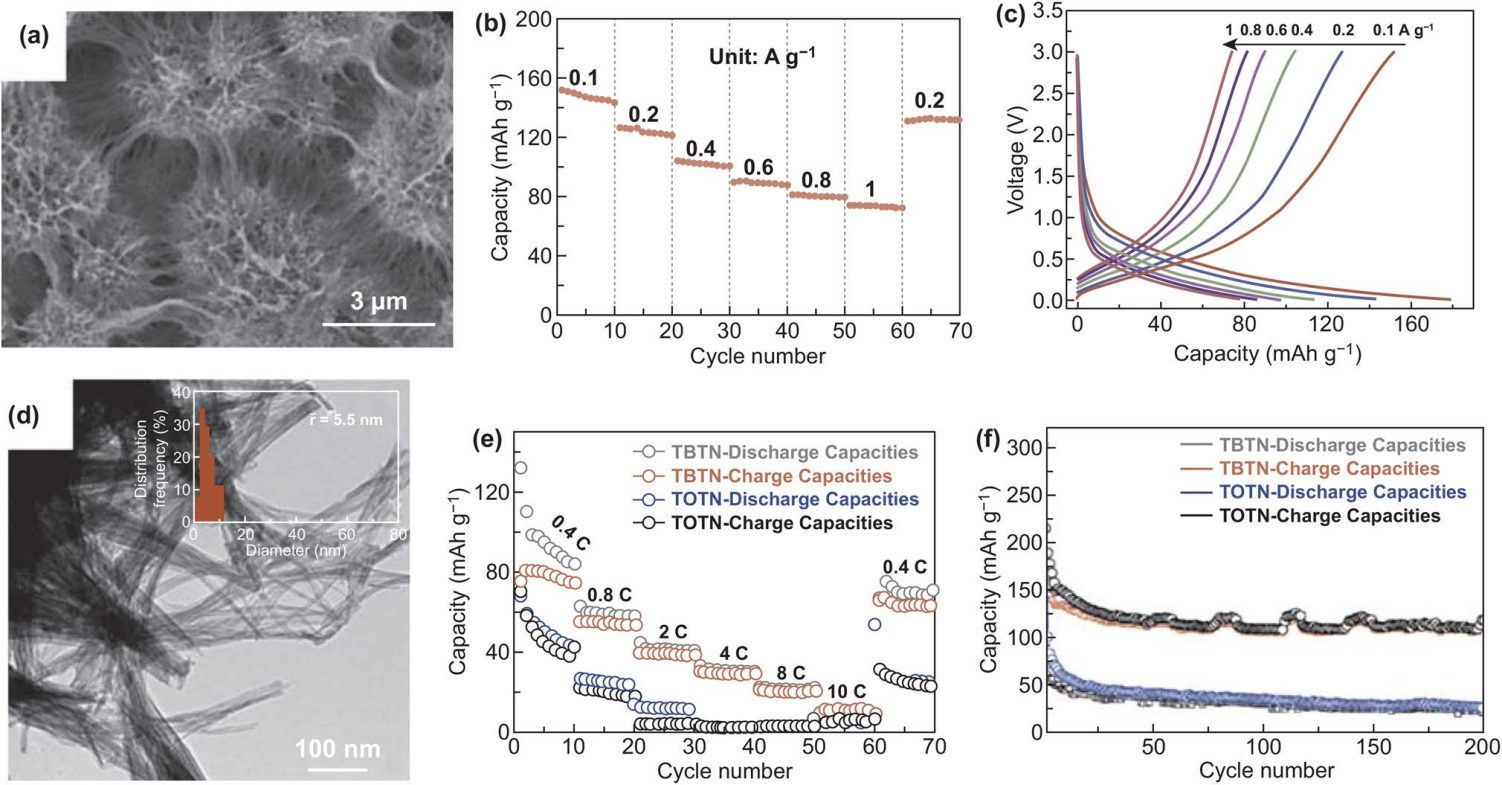
**a** T-Nb_2_O_5_’s SEM image. **b** T-Nb_2_O_5_ anode’s rate performance in the range of 0.1–1 A g^−1^. **c** Corresponding charge capacities under various current densities. Reproduced with permission from Ref. [228]. Copyright 2018, Royal Society of Chemistry. **d** TEM image of TBTN with average diameter of around 5.5 nm. **e** Rate performance and **f** cycle performance of TBTN. Reproduced with permission from Ref. [71]. Copyright 2018, John Wiley and Sons

3D MCs and MOs have aroused increasing attentions in PIBs electrode materials. For example, in 2017, it was the first time that Ren et al. [70] used MoS_2_ particles as electrode materials in PIBs. The author indicated that the micro-sized MoS_2_ with layer structure was beneficial to stably form K-ion intercalation compound (K_0.4_MoS_2_) during potassiation to 0.5 V. It is demonstrated that MoS_2_ may be considered as another choice of PIBs anode materials. Besides, increasing 3D metal-based oxides have been tried for PIBs anode. In 2018, Li and co-workers [228] firstly introduced orthorhombic niobium pentoxide (T-Nb_2_O_5_) into PIBs anode field. The urchin-like interlinked hierarchical structure of T-Nb_2_O_5_ assembled by nanowires was exhibited (Fig. 13a), which could boost K-ion diffusion. Therefore, the capacity of 104 mAh g^−1^ was delivered at 0.4 A g^−1^ in terms of rate performance (Fig. 13b, c). In addition, interconnected K_2_Ti_6_O_13_ nanowires framework was fabricated as electrode materials in PIBs [71]. The K_2_Ti_6_O_13_ nanowires with a diameter of around 5.5 nm (name as TBTN) were displayed (Fig. 13d). In this framework, ionic diffusion and mechanical robustness could be enhanced due to 3D interlinked architectures. Thus, the TBTN electrode delivered the rate capacities of 11 mAh g^−1^ at 10 C (Fig. 13e). Besides, the cycle capacity of around 120 mAh g^−1^ was exhibited at 0.2 C after 20th cycle (Fig. 13f). From the above analysis, it can be concluded that good structural design helps to improve the performances of 3D MCs and MOs, but these properties are not satisfactory enough. Judging from the characteristics of these materials, the reason may put down to their poor conductivity. Therefore, it is necessary for 3D MCs and MOs to combine other modification methods to obtain satisfactory electrochemical performances.

### Three-Dimensional Alloying Materials

**Fig. 14:**
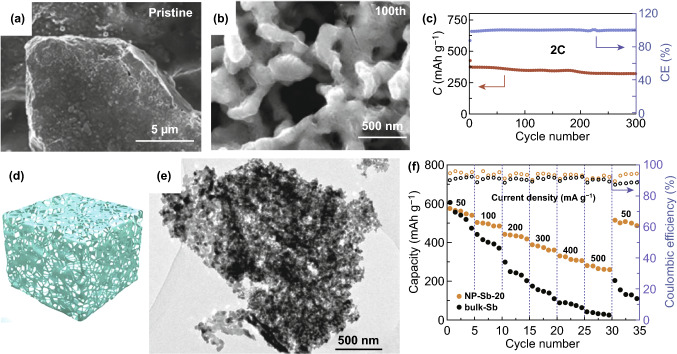
SEM image of **a** pristine Bi and **b** Bi after 100th cycle. **c** Cycle performance of Bi electrode (1 C = 384.7 mAh g^−1^). Reproduced with permission from Ref. [229]. Copyright 2018, John Wiley and Sons. **d** Schematic illustration of NP-Sb. **e** TEM image of NP-Sb-20. **d** Rate performance of two samples in the range of 50–500 mA g^−1^. Reproduced with permission from Ref. [72]. Copyright 2018, American Chemical Society

3D alloying materials are beneficial to contact largely with electrolyte and buffer the volume expansion, especially 3D porous structures. Pristine Bi block was used as PIBs anode and would gradually transform into 3D porous networks after 100 cycles during charge and discharge process (Fig. 14a, b) [229]. The formed porous networks not only enhanced ionic transfer but also restricted the stress variation in the course of potassium/depotassium process. Therefore, the capacity retention realized 86.9% after 300 cycles at 2 C (Fig. 14c). This method provides new ideas for constructing porous alloying materials. So nanoporous Sb (NP-Sb) was synthesized as PIBs anode materials (Fig. 14d) [72]. The pores of NP-Sb-20 (20 means the percentage of Sb atom) were uniformly distributed in the continuous porous structure (Fig. 14e). Benefiting from the 3D porous architecture, the NP-Sb-20 could effectively tolerate stress variation and promote ions transportation. As for rate performance, the bulk Sb only delivered 30 mAh g^−1^ at 500 mA g^−1^, while the NP-Sb-20 delivered 265 mAh g^−1^ (Fig. 14f). Obviously, the performances improvement was due to the nanoporous structure of Sb. Besides, in 2019, nanoporous Ge was fabricated for the first time, which delivered the capacity of around 120 mAh g^−1^ at 20 mA g^−1^ over 400 cycles [73]. Based on nanoporous Ge with the numerous pores as well as nanoscale ligaments, the stable cycling performance was ascribed to shorter diffusion distance of K-ion as well as the adequate space for volumetric variation. Overall, 3D porous structures are of benefit to alloying materials and further efforts should be made to improve the electrochemical behavior.

Additionally, given the discussion about multi-dimensional structures, related nanomaterials possess attractive advantages and unfavorable disadvantages, so optimized strategies should be taken to improve their electrochemical performances. Accordingly, the corresponding advantages, disadvantages, and optimized strategies are outlined in Table 5.

**Table 5:** Advantages, disadvantages, and optimized strategies of multi-dimensional nanostructures

Materials	Advantages	Disadvantages	Optimized strategies	References
0D nanomaterials	Sufficient sites for ionic adsorption; buffering large volume expansion; shorter ionic diffusion pathway	Easy aggregation; low tap density	Combining with carbon materials; compounding with micro-sized materials	[230–234]
1D nanomaterials	High mechanical robustness; facilitating electronic and ionic transfer	Nonadjustable specific surface area and porosity properties	Coating; second phases used to decoration; forming the interconnected network	[235–237]
2D nanomaterials	Large surface area; enhanced open-edge morphologies; high conductivity; tunable interlayer spacing; relieving the stress changes	Side reactions; easy agglomeration	Surface coating; compounding with other materials; doping with other heteroatoms	[238–243]
3D nanomaterials	Inhibition of agglomeration; free-standing; high mechanical stability; tolerating the stress variation; facile ionic access	Numerous macropores affecting the capacitive-controlled contribution; low initial Coulombic efficiency	Combining with low-dimensional materials; surface engineering	[225, 244–249]

## Summary and Outlook

As aforementioned, PIBs have been investigated due to better advantages compared with LIBs and SIBs, such as abundant resources, lower price, and smaller stokes radius of solvated ions. However, there are still some obstacles to PIBs for commercial applications, such as large volumetric variation induced by large size of K-ion during charge and discharge process, weak ionic diffusivity in solid phase, inferior kinetics of K^+^ reaction, the existence of side and irreversible reactions, the imperfect energy store mechanism compared with LIBs, the production of dendrite and related safety problems. Accordingly, nanostructural design has been considered as one of the effective strategies to enhance corresponding performances.

In summary, 0D–3D nanomaterials about PIBs anode materials have been summarized and involved in the relationship with the corresponding electrochemical performances, mainly concerning carbon materials, MCs and MOs, and alloying materials. 0D nanomaterials have been utilized in PIBs anode materials due to nano-size and large surface area, which can boost ionic transportation and alleviate the stress changes. In addition, 1D nanomaterials with high length-to-diameter aspect ratios possess high mechanical robustness as well as shorter electronic and ionic transport path. Moreover, 2D nanomaterials have also possessed large surface area to enhance ionic adsorption and diffusion in PIBs anode materials field. Finally, the interconnected structure about 3D nanomaterials can make largely electrode and electrolyte contact, which can facilitate ionic transfer.

Given aforementioned information, 0D–3D nanomaterials possess different structural and morphological features, corresponding to different electrochemical performances. Therefore, in order to better understand the different effect of multi-dimensional nanomaterials, it is necessary to make a comparison about their electrochemical performances. Furthermore, the corresponding comparision would be discussed by taking 0D–3D carbon materials as examples. And then, typical carbon nanomaterials will be introduced, including carbon nanocage, CNTs, graphene, and graphite. Among them, 0D carbon nanocage possesses large surface area and unique cage-like structure, which makes it have well electrochemical performances (e.g., 195 mAh g^−1^ at 0.2 C after 100 cycles for CNC) [42]. Additionally, as representative 1D carbon nanomaterial, compared with 0D nanocage, CNTs with high aspect ratio are conducive to enhance the mechanical strength of electrode materials. So the structure of related electrode can be maintained stable and durable during charging and discharging, improving the long cycling life (e.g., 244 mAh g^−1^ at 0.5 A g^−1^ after 200 cycles for P@TBMC-2.4) [50]. As for graphene, with large landing platform, they are beneficial for adsorbing K-ion and are different from 0D as well as 1D carbon materials, which can evidently improve the capacity by adsorption mechanism (e.g., 385 mAh g^−1^ at 500 mA g^−1^ after 600 cycles for PODG) [66]. Moreover, graphite can be used to inhibit the aggregation as a comparison of 0D–2D nanomaterials, which can keep structure stable and retain the stable capacity (e.g., 174 mAh g^−1^ at 200 mA g^−1^ after 500 cycles for expanded graphite) [109]. According to the comparison and discussion of 0D–3D carbon materials, the difference of electrochemical performances of multi-dimensional nanomaterials can be well understood. Additionally, the synthetic methods are important to prepare nanostructures with excellent electrochemical performances. Therefore, the main preparation methods about their advantages and disadvantages are summarized in Table 6. From the results of comparison, Ball milling may be considered as the practical strategies to obtain nanomaterials in the industries, which is attributed to its facile operability, low cost, and large scale.

**Table 6:** Summary of advantages and disadvantage of various synthesis methods for multi-dimensional structures

Synthesis methods	Advantages	Disadvantages	References
Ball milling method	Facile; highly effective; economic; scalable	Products with uneven and large size; generation of noise	[33, 40, 45, 48, 250]
Hydrothermal/solvothermal method	Facile morphological control; controllable size; high purity	High cost; simple morphology; difficult controlled processes; existing safety problems	[38, 46, 61, 62, 99, 106, 157, 199]
Solution method	Well-controlled size; uniform dispersion	Related toxic reagent	[47, 63, 95, 165, 214, 251, 252]
Vaporization–condensation method	Uniform and small size; controllable nanostructure	Difficult controlled distribution; low production efficiency	[50, 122, 217, 253]
Calcination/carbothermic reduction	Well operability; generation of new phases	Large energy consumption; generation of toxic gases	[44, 53–55, 57, 80, 81, 89, 96–98, 100–102, 104, 105, 128, 129, 131, 132, 135, 198, 202]
Electrodeposition method	Uniform size; controllable	Related toxic reagent; large energy consumption	[49]
Solid–liquid reaction method	Simple; facile operability	Related dangerous solution and toxic reagent	[52, 60, 103, 254–256]
Chemical vapor deposition method	Precise controlled products; high purity	Required relatively high deposition temperatures; involving some dangerous precursors; large energy consumption	[127, 257]

Although significant progress has been achieved due to nanostructure design with different dimensions, more efforts should be made for PIBs anode materials to improve electrochemical performances as followed:

1. Novel architecture should be proposed. For example, carbon dots have been directly explored for LIBs and SIBs, while carbon dots only as raw materials were used to fabricate PIBs anode materials. Thus, further experiments may focus on novel structure design.
2. Novel synthetic strategies about different dimensional structures like 3D printing method can be used in PIBs field. Therefore, it is necessary to create methods with simple process and low cost.
3. Different dimensional structures can be assembled to fabricate various materials with unique morphology to sufficiently achieve the properties of every component.
4. Using new materials to design different dimensional structures is beneficial for increasing the variety of anodes as well as trying to improve the electrochemical performances.
5. The relationship between different dimensional structural electrodes and various electrolytes should be studied. To date, a few studies have involved in the impact of electrolytes on corresponding performances.
6. Some energy store mechanisms of PIBs still keep unclear, so more efforts should be undertaken.

All in all, it is one of the key preconditions for commercial PIBs anode materials to utilize simple preparation methods and simplified processes in different dimensional materials fabrication. Meanwhile, high-performance, low-cost, and good-stability materials will be considered as the desire choice in the practical application. Therefore, this review is devoted to provide new insights for further research.
